# Phase 1, randomized trials of MEDI1341: cerebrospinal fluid free α-synuclein lowered by >50%

**DOI:** 10.1093/braincomms/fcaf304

**Published:** 2025-08-19

**Authors:** Craig Shering, Michael Pomfret, Robert J Kubiak, Isabelle J Pouliquen, Wei Yin, Arthur Simen, Elena Ratti, Zubair Hussain, Michael Perkinton, Keith Tan, Kirsten M Scott, Thor Ostenfeld, Iain Chessell

**Affiliations:** Neuroscience, BioPharmaceuticals R&D, AstraZeneca, Boston, MA 02210, USA; Neuroscience, BioPharmaceuticals R&D, AstraZeneca, Trumpington, Cambridge CB2 0AA, UK; Clinical Pharmacology & Safety Sciences, BioPharmaceuticals R&D, AstraZeneca, Gaithersburg, MA 20878, USA; Clinical Pharmacology & Safety Sciences, BioPharmaceuticals R&D, AstraZeneca, Trumpington, Cambridge CB2 0AA, UK; Department of Quantitative Clinical Pharmacology, Takeda Development Center Americas Inc., Cambridge, MA 02142, USA; Neuroscience Therapy Area Unit, Takeda Development Center Americas Inc., Cambridge, MA 02142, USA; Neuroscience Therapy Area Unit, Takeda Development Center Americas Inc., Cambridge, MA 02142, USA; Neuroscience, BioPharmaceuticals R&D, AstraZeneca, Trumpington, Cambridge CB2 0AA, UK; Neuroscience, BioPharmaceuticals R&D, AstraZeneca, Trumpington, Cambridge CB2 0AA, UK; Neuroscience, BioPharmaceuticals R&D, AstraZeneca, Trumpington, Cambridge CB2 0AA, UK; Neuroscience, BioPharmaceuticals R&D, AstraZeneca, Trumpington, Cambridge CB2 0AA, UK; Neuroscience, BioPharmaceuticals R&D, AstraZeneca, Trumpington, Cambridge CB2 0AA, UK; Neuroscience, BioPharmaceuticals R&D, AstraZeneca, Trumpington, Cambridge CB2 0AA, UK

**Keywords:** Parkinson’s disease, CSF, safety, pharmacokinetic, pharmacodynamic

## Abstract

Accumulation of pathological forms of α-synuclein is a hallmark of Parkinson’s disease. MEDI1341 (also known as TAK-341) is a high-affinity, α-synuclein-specific, fully human monoclonal antibody that binds the C-terminal region of human α-synuclein. Pre-clinical studies of MEDI1341 demonstrated significant reductions in α-synuclein accumulation and propagation along axons. Two randomized, double-blind, placebo-controlled, Phase 1 studies administering MEDI1341 intravenously were conducted; a single ascending dose study (NCT03272165) of MEDI1341 (70, 210, 400, 1200, 2400 or 4500 mg; *n* = 6 each) or placebo (*n* = 13 total) in healthy participants and a multiple ascending dose study (NCT04449484) of 4-weekly MEDI1341 (1200 or 2000 mg; *n* = 9 each) or placebo (*n* = 7 total) in participants with Parkinson’s disease. Both studies assessed the safety and tolerability of MEDI1341 versus placebo, with MEDI1341 pharmacokinetics, pharmacodynamics and immunogenicity as secondary objectives. Pharmacokinetic (MEDI1341 in serum and CSF) and pharmacodynamic (total α-synuclein in plasma, and free α-synuclein in CSF) concentrations were determined using validated electrochemiluminescence assays. Overall, 49 healthy participants (67.3% male; mean [standard deviation] age 43.4 [9.5] years) were included in the single ascending dose study and 25 participants with Parkinson’s disease (72.0% male; mean [standard deviation] age 63.0 [9.0] years; 88% Hoehn and Yahr stage 2) were included in the multiple ascending dose study. Treatment-emergent adverse events were reported in 23 healthy participants (MEDI1341, *n* = 17; placebo, *n* = 6) and 10 participants with Parkinson’s disease (MEDI1341, *n* = 9; placebo, *n* = 1). The most common treatment-related treatment-emergent adverse events were headache, fall and nausea. Dose proportional increases were observed for maximum concentration in serum and area under the curve (AUC) in both studies, with the exception of a supra-proportional increase in AUC_0–∞_ from 2400 to 4500 mg (single ascending dose study). Median time to maximum concentration was 1 h after intravenous administration and geometric mean terminal elimination half-life ranged from 16.6 to 24.3 days across both studies. Suppression of α-synuclein in CSF was greatest at the highest doses investigated: −53.6% median change from baseline on Day 15 [4500 mg (healthy participants)] and −59.0% median change from baseline on Day 85 [2000 mg (participants with Parkinson’s disease)]. Across all doses and time points, individual participant CSF MEDI1341 concentrations were <1% of their respective serum concentrations. MEDI1341 had favourable safety, tolerability, pharmacokinetic and pharmacodynamic profiles in healthy participants and those with Parkinson’s disease, supporting further clinical development. MEDI1341 is the first monoclonal antibody targeted against α-synuclein to demonstrate a > 50% reduction in CSF-free α-synuclein.

## Teaser

MEDI1341 is a human monoclonal antibody targeting α-synuclein (a key protein in Parkinson’s disease) with high affinity/specificity. Phase 1 trials by Shering *et al.* report MEDI1341 reduced free α-synuclein in CSF by >50% and was generally well tolerated. Whilst promising, the effect on symptoms needs further study.

## Graphical Abstract

**Graphical Abstract:**
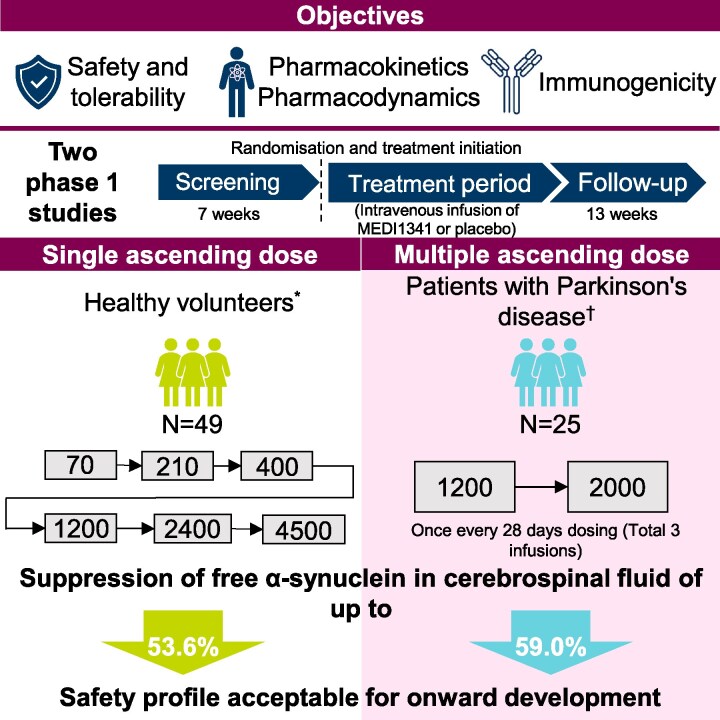

## Introduction

The accumulation of pathological forms of the protein α-synuclein is the hallmark of a group of disorders known as synucleinopathies.^1^ These disorders include multiple system atrophy, pure autonomic failure, rapid eye movement sleep behaviour disorder, Parkinson’s disease and Lewy body dementia. Multiple system atrophy is unique amongst these disorders as it is characterized histologically by the accumulation of aggregated α-synuclein, primarily in oligodendroglia (glial cytoplasmic inclusions) rather than in neurons.^2^ Clinically, multiple system atrophy is characterized by Parkinsonism, ataxia, and severe autonomic dysfunction, and patients with its more aggressive course have a 45% chance of experiencing falls at least once a day and a 15% chance of unintelligible speech within 2 years of diagnosis.^3^ It may be preceded by rapid eye movement sleep behaviour disorder. Other synucleinopathies, including Parkinson’s disease, are characterized histologically by Lewy bodies and Lewy neurites—neuronal intracellular inclusions of misfolded aggregated protein, mainly α-synuclein fibrils.^4^

Parkinson’s disease is defined clinically by tremor, bradykinesia and rigidity, but its course is also associated with autonomic dysfunction and dementia,^5-7^ with symptoms reflecting progressive neurodegeneration.^7,8^ It is characterized by the loss of dopaminergic cells in the substantia nigra pars compacta. Subsequent spread of pathology, including to the cortex or autonomic nervous system, is associated with cognitive impairment or autonomic dysfunction, respectively.^7^ Cell death and neuroinflammation occur in regions with a higher pathological burden.^1^ Given the established genetic link to *SNCA* (the gene encoding α-synuclein) in Parkinson’s disease, rapid eye movement sleep behaviour disorder and Lewy body dementia,^8-12^ there has been a suggestion to shift from a clinical to a biological definition of Parkinson’s disease (and dementia with Lewy bodies). The recently proposed neuronal α-synuclein disease integrated staging system defines Parkinson’s disease biologically by the presence of pathological α-synuclein, dopaminergic neuron dysfunction, and pathogenic variants in the *SNCA* gene.^13^

At present, there are no disease-modifying therapies available for synucleinopathies,^8^ and the current standard of care focuses on symptom management. Given the high disease burden for patients and their families resulting from progressive loss of independence and function in aspects such as mobility, communication, cognitive function and swallowing, there is an urgent unmet need for therapies that can slow disease progression.^8^

A common pathogenic hypothesis proposed across all synucleinopathies is the ‘seeding’ of pathological forms of α-synuclein, which subsequently spread through the nervous system, resulting in disease progression.^14^ In the healthy brain, α-synuclein exists as unfolded monomers;^15^ however, in synucleinopathies, the balance between generation and clearance of α-synuclein is disrupted, and the monomers aggregate to form oligomers, which are thought to be the toxic species.^15,16^ Further aggregation results in the formation of protofibrils and fibrils, contributing to the characteristic Lewy body pathology observed in patients with Parkinson’s disease.^15^ Recent investigations into the pathological spread of α-synuclein in synucleinopathies have suggested both ‘brain-first’ and ‘body-first’ models; pathology spreads either from the brain to the peripheral autonomic nervous system, or from the body (specifically the enteric and peripheral autonomic nervous systems) to the brain.^17^ The hypothesis that pathological α-synuclein is transmitted through the nervous system arose from the post-mortem identification of Lewy body pathology 10 years after transplantation of embryonic mesencephalic neuronal grafts.^18^ This was further explored by Braak *et al.* in the pathological staging of Parkinson’s disease, which assumes sequential spread from region to region.^19^

Owing to its proposed key role, α-synuclein is one of the most compelling targets for the development of disease-modifying therapies targeting synucleinopathies.^20-22^ Immunotherapy with monoclonal antibodies targeting extracellular α-synuclein may prevent pathological spreading of α-synuclein, slowing the progression of Parkinson’s disease, multiple system atrophy and other synucleinopathies.^23,24^ Pre-clinical models in mice have shown that passive immunization is effective at removing pathological α-synuclein and improving related deficits.^20-22^

Antibodies targeting α-synuclein have been studied in the context of Parkinson’s disease. Phase 2 trials of prasinezumab and cinpanemab in early Parkinson’s disease showed no benefit over placebo in terms of disease progression, but an exploratory analysis showed that prasinezumab may reduce motor progression to a greater extent over the course of a year in individuals with more rapidly progressing Parkinson’s disease.^25-29^ The reason for the lack of demonstrated efficacy of antibodies targeting α-synuclein in Parkinson’s disease is unclear but may be due to antibody design, for example, targeting the wrong epitope, low specificity, or low affinity to monomeric or aggregated α-synuclein.^30^ The lack of efficacy during the prasinezumab trial may have been due to slow disease progression overall, making it difficult to detect a between-group difference. However, *post hoc* analysis suggests that enriching for patients progressing at a faster rate may allow the identification of an efficacy signal at an earlier time point.^26^

The published literature does not allow for a clear assessment of target engagement in the CNS with either prasinezumab or cinpanemab. Ensuring adequate CNS target engagement is critical, as it is a common reason for a lack of efficacy in clinical drug development, as the major limitation of monoclonal antibody approaches is that they can only target extracellular α-synuclein. Accordingly, it is unclear whether this approach is sufficient to modulate the pathology of Parkinson’s disease, as small-molecule approaches may allow for greater removal of α-synuclein. The results from the previous trials of α-synuclein targeting therapies do not rule out α-synuclein as a target in these diseases.

MEDI1341 is a high-affinity, α-synuclein-specific, fully human IgG1 monoclonal antibody, binding specifically to the C-terminal region of human α-synuclein.^31^ It is engineered for reduced effector function by virtue of a triple mutation introduced into the fragment crystallizable region of the antibody.^31^ MEDI1341 can distribute into the brain and binds both the monomeric and aggregated forms of α-synuclein, sequestering extracellular α-synuclein and reducing its spread through the brain, which is hypothesized to slow or halt the progression of α-synucleinopathies, including Parkinson’s disease and multiple system atrophy. During the development of MEDI1341, it was hypothesised that removal of all species of α-synuclein, both monomeric and aggregated forms, rather than targeting aggregated forms alone, would be more effective at reducing the effect of toxic oligomers by preventing misfolding, seeding, and early propagation in addition to neutralising toxic aggregates of α-synuclein. This effect has been shown in pre-clinical studies of MEDI1341, which demonstrated the attenuation of cell-to-cell spreading of α-synuclein pathology in the mouse brain.^31^ Moreover, MEDI1341-mediated reduction of free α-synuclein concentrations in CSF has been demonstrated in pre-clinical studies of rats and monkeys, and reduction of free α-synuclein in brain interstitial fluid has been observed in rats.^31^ MEDI1341 has been shown to bind to α-synuclein in post-mortem brain samples from patients with both Parkinson’s disease and multiple system atrophy in a typical staining pattern reflecting Lewy Body pathology or glial cytoplasmic inclusions (respectively). Based on this evidence, differences in the conformation of α-synuclein between Parkinson’s disease and multiple system atrophy (e.g. as revealed by cryo-EM) are unlikely to affect the efficacy of the antibody.^32^

Two studies were undertaken to evaluate the safety, tolerability, pharmacokinetics, pharmacodynamics and immunogenicity of single and multiple ascending infusions of MEDI1341 in healthy participants and participants with Parkinson’s disease, respectively. These studies form the first stage in a development program to evaluate whether targeting extracellular α-synuclein with MEDI1341 slows the clinical progression of synucleinopathies.

## Materials and methods

### Study design

**Figure 1:**
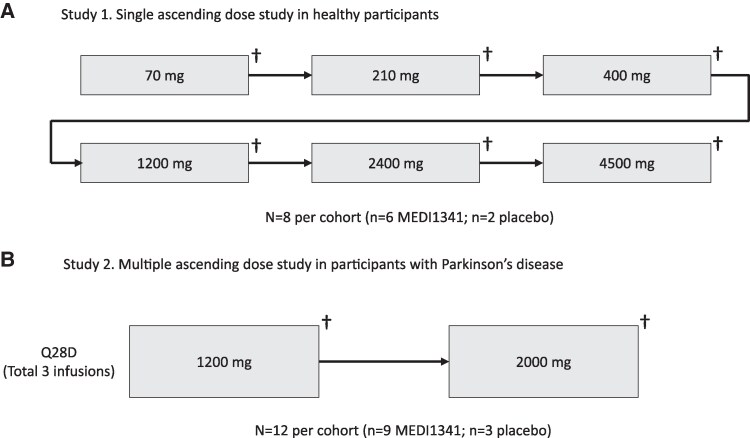
Design of the (**A**) single ascending dose study in healthy participants and (**B**) multiple ascending dose study in participants with Parkinson’s disease. †Dose escalation committee review. Q28D, once every 28 days.

The development programme included two phase 1 randomized, double-blind, multicentre, placebo-controlled studies of MEDI1341 administered as a single intravenous infusion to healthy participants (study 1) and repeat 4-weekly intravenous infusions (three occurrences) to participants with Parkinson’s disease (study 2). Study 1 (NCT03272165) was a single ascending dose (SAD) study of intravenous MEDI1341 (70, 210, 400, 1200, 2400 or 4500 mg) or placebo [0.9% (w/v) sodium chloride] administered to healthy participants (Fig. 1) at two centres in the USA. It was conducted between 26 October 2017 and 31 March 2021. The study comprised a screening period of up to 7 weeks, a double-blind treatment period during which a single 60-minute intravenous infusion of MEDI1341 or placebo was administered, and a 13-week follow-up period. Study 2 (NCT04449484) was a multiple ascending dose (MAD) study of intravenous MEDI1341 (1200 or 2000 mg) or placebo (0.9% [w/v] sodium chloride) administered every 28 days (on days 1, 29 and 57) as a 60-minute intravenous infusion to participants with Parkinson’s disease (Fig. 1). The study was conducted at six centres in the US and comprised a 7-week screening period, a 12-week double-blind treatment period, and a 13-week follow-up period. It was conducted between 4 August 2020 and 5 January 2022. Due to the exploratory nature of the SAD and MAD studies, sample size was based primarily on a desire to obtain sufficient safety and tolerability, as well as pharmacokinetic/pharmacodynamic information, while exposing as few subjects as possible to the investigational treatment rather than on formal statistical considerations.

For both studies, a Dose Escalation Committee reviewed the safety, tolerability and pharmacokinetic data from each cohort, as well as available pharmacodynamic and antidrug antibody (ADA) data, before progression to the next higher dose cohort was allowed. The studies were conducted in accordance with the Declaration of Helsinki, the Council for International Organizations of Medical Sciences International Ethical Guidelines and the International Council for Harmonisation Good Clinical Practice Guideline. The studies were also conducted in accordance with local legal and regulatory requirements and with standard operating procedures in place. Prior to study initiation, the respective study protocol, informed consent form, and other relevant documents were reviewed and approved by the appropriate independent ethics committee/institutional review board. All amendments to the respective protocol were approved by the institutional review board. Written informed consent was obtained from participants or their legal representative prior to any study procedures.

### Study population

Owing to the different populations evaluated, eligibility criteria were different for the two studies. For the SAD study, healthy males or females of non-childbearing potential aged 18–65 years with a body weight ≥50 kg and body mass index (BMI) 18.0–32.0 kg/m^2^ were eligible for inclusion. Concomitant medications, with the exception of paracetamol and contraceptives, were not permitted until the end of the follow-up period unless required in the opinion of the Investigator for the participant’s safety and well-being. Prior use of prescription or non-prescription medication, including aspirin, vitamins, or herbal or dietary supplements, within 7 days or five half-lives (whichever was longer) of intravenous MEDI1341 administration was not permitted. Full inclusion and exclusion criteria for the SAD study are listed in the Supplementary material.

For the MAD study, male and female participants of non-childbearing potential aged 40–85 years with a diagnosis of mild-to-moderate idiopathic Parkinson’s disease according to the United Kingdom Parkinson’s Disease Society Brain Bank criteria (stage 1 to 3 according to the modified Hoehn and Yahr scale), body weight 40–120 kg, BMI 18.0–34.0 kg/m^2^ and a stable dosing regimen of their medication for ≥1 month before randomization (with no expectation of a need to change the medications or dosing regimen for the duration of the study, barring unforeseen circumstances) were eligible for inclusion. Any standard of care therapy appropriate for the management of Parkinson’s disease was permitted, with the exception of those listed in the exclusion criteria (see Supplementary material). Any other medications considered necessary for the participant’s safety and well-being were given at the discretion of the Investigator. Key exclusion criteria included recent or present clinically significant illness, prior/concomitant therapy with a monoclonal antibody, antipsychotic or immunosuppressive medication within 6 months, or investigational medicine, device or biologic within 3 months or five half-lives of the intervention, and any clinically significant abnormalities unrelated to Parkinson’s disease. Full inclusion and exclusion criteria for the MAD study are listed in the Supplementary material.

### Objectives

For both studies, the primary objective was to assess the safety and tolerability of MEDI1341 versus placebo. The secondary objectives were to assess the pharmacokinetics, pharmacodynamics and immunogenicity of MEDI1341. The effect of MEDI1341 compared with placebo on free and total α-synuclein was also assessed as an exploratory objective in both studies.

### Procedures

In the SAD study, six participants were randomized to receive MEDI1341 and two to receive a placebo within each of the six dose cohorts of eight participants. Within each dose cohort of twelve participants in the MAD study, nine were randomized to receive MEDI1341 and three to receive a placebo. Full randomization procedures are reported in the Supplementary material.

Eligible participants received an approximately 60-minute intravenous infusion of MEDI1341 or placebo in a double-blind manner on Day 1 (SAD) or Days 1, 29 and 57 (MAD). Personnel preparing the study drug, responsible for expedited safety reporting, analysing the pharmacokinetic, pharmacodynamic and immunogenicity samples, and preparing summaries for the Dose Escalation Committee had access to treatment assignments. The Dose Escalation Committee reviewed unblinded data. All other personnel, including the participants, investigators, sponsor study team and its delegates not part of the Dose Escalation Committee, remained blinded to the treatment for the duration of the respective study. As MEDI1341 and placebo were distinguishable in appearance, an unblinded intervention manager prepared MEDI1341 for infusion and covered the infusion bag with a coloured sleeve.

Participant safety was monitored throughout the study. Adverse events (AEs) that were on-going at the time of discontinuation from the study were followed for up to 30 days or until the Investigator considered the condition to be stable. Any therapies administered to treat an AE were recorded. Other safety evaluations included vital signs (supine and standing blood pressure, pulse rate and body temperature), electrocardiograms, physical and neurological examinations, and safety laboratory tests (haematology, clinical chemistry, coagulation, urinalysis) (see Supplementary Tables 1 and 2**)**.

Free α-synuclein was defined as the monomeric, unaggregated form of the protein that is not bound to MEDI1341 or incorporated into aggregates. Total α-synuclein was defined as the sum of free α-synuclein and α-synuclein bound to MEDI1341.

CSF samples were taken via lumbar puncture to determine CSF concentrations of MEDI1341 and free α-synuclein. As the optimum time points for pharmacodynamic profile assessment were unknown prior to SAD study initiation, the timing of lumbar puncture was modified throughout the study, based on the review of aggregated pharmacokinetic and pharmacodynamic data as part of the dose escalation review process. One lumbar puncture was performed the day before dosing and one or two were performed on either Day 8, Day 15 or Day 29 in the SAD study. Lumbar punctures were performed the day before first dosing, on Day 61 and Day 85 in the MAD study.

Blood and CSF samples were collected for the determination of pharmacokinetic and pharmacodynamic concentrations at specific time points (Supplementary Tables 1 and 2). Validated electrochemiluminescence assays were used to quantitatively determine MEDI1341 concentrations in human serum and CSF, total α-synuclein concentrations in plasma, and free α-synuclein concentrations in CSF (Supplementary material). To avoid potential variability due to differences in operator and assay lot over time, the CSF samples for free α-synuclein concentration determination were reanalysed using the same assay kit on completion of all cohorts in the SAD. The analyst was blinded to the assay plate map in both studies.

Blood samples to evaluate serum ADAs were collected at specific time points (Supplementary Tables 1 and 2) and as soon as possible following a serious AE (SAE) in both studies and were analysed using a validated electrochemiluminescence assay. ADA results were classified as negative, borderline positive or positive (Supplementary material). Positive and borderline positive results were assessed for their impact on pharmacokinetics and pharmacodynamics.

Comprehensive ophthalmic assessments (including optical coherence tomography) were added to the SAD study design and included in the MAD study based on observations of scleral thickening and mononuclear infiltrates in the corneo-scleral junction during a pre-clinical study in rats (Supplementary material).^31^

Disease stability was assessed in participants with Parkinson’s disease using the Movement Disorder Society—Unified Parkinson's Disease Rating Scale (MDS–UPDRS) at screening, Day 85 (end of treatment) and Day 148 (during follow-up), with changes from baseline calculated and presented for total scores and for Parts I–IV summarized by treatment type. Montreal Cognitive Assessment (MoCA) was performed the day before the first infusion of MEDI1341, and at Days 85 and 148. There was no expectation to detect a clinical effect due to the small sample size and the short duration of treatment.

### Statistical analysis

TEAEs were reported according to the number and percentage of participants they occurred, and summarized by treatment, severity, and relationship to MEDI1341. TEAE frequency was reported as the number of events, and summarized by treatment, system organ class and preferred term. Injection site and infusion reactions were summarized as numbers and percentages by time point.

MEDI1341 serum and CSF concentrations and/or derived pharmacokinetic parameters were summarized by treatment regimen using descriptive statistics. Maximum observed serum concentration of MEDI1341 (C_max_), area under the concentration–time curve from time zero to infinity [AUC _0–∞_ (SAD study only)], area under the concentration–time curve from time zero to the last quantifiable concentration (AUC_0-t_), area under the concentration–time curve from time zero to the end of the dosing interval (AUC_0-τ_), terminal elimination half-life of MEDI1341 (t_1/2λz_; after last dose in MAD study), total serum clearance (CL; after last dose in MAD study), volume of distribution during the terminal elimination phase (V_z_; after last dose in MAD study); and volume of distribution at steady-state [V_ss_ (SAD study only)] were reported as geometric mean [coefficient of variation (CV%)]. Time of maximum observed serum concentration of MEDI1341 (t_max_) was reported as median (range). MEDI1341 concentrations from CSF were summarized as percentages of serum concentrations and geometric mean CSF/serum concentration ratios were reported across dose levels. Between-participant variability was assessed using geometric CV for continuous pharmacokinetic variables. Dose proportionality and linearity were assessed in both studies (see Supplementary material)

Changes from baseline in plasma total α-synuclein were summarized as absolute mean (SD) change and mean (SD) and median (range) percentage changes. Mean (SD) and median (range) percentage change from baseline in CSF-free α-synuclein were also derived. MoCA and MDS–UPDRS total scores for Part I–IV were summarized by treatment and time point as mean (SD) change from baseline. Pre-specified analyses were carried out using SAS (version 9.4 or higher) using pre-specified shells.

Exploratory *post hoc* statistical analyses were conducted to determine whether MEDI1341 doses had a significant effect on free α-synuclein versus placebo using pairwise Welch’s *t*-tests. This exploratory statistical analysis was carried out using R (Version 2023.12.1 + 402) using the lme4, dplyr and emmeans packages. The significance of differences in baseline demographics and clinical characteristics between treatment groups was also assessed *post hoc* using the Wilcoxon rank sum test.

## Results

### Demographics and baseline characteristics

A total of 49 healthy participants were eligible for inclusion in the SAD study (MEDI1341, *n* = 36 [*n* = 6 for each dose cohort]; placebo, *n* = 13 total). Participants were mostly male (67.3%) and predominantly white (49.0%), with a mean (SD) age of 43.4 (9.5) years, mean (SD) BMI of 27.3 (2.8) kg/m^2^ and mean (SD) weight of 80.1 (12.5) kg. Demographics and baseline characteristics were comparable across the cohorts and between those receiving MEDI1341 and placebo (**Table 1**).

**Table 1:** Baseline demographics and characteristics of healthy participants in the single ascending dose study

	Placebo (*N* = 13)	70 mg (*N* = 6)	210 mg (*N* = 6)	400 mg (*N* = 6)	1200 mg (*N* = 6)	2400 mg (*N* = 6)	4500 mg (*N* = 6)	Overall (*N* = 49)
Age, years, mean (SD)	42.5 (9.1)	47.8 (5.0)	37.0 (6.3)	39.0 (12.6)	45.3 (12.7)	48.0 (5.3)	45.2 (11.1)	43.4 (9.5)
Male sex, *n* (%)	8 (61.5)	4 (66.7)	5 (83.3)	4 (66.7)	2 (33.3)	6 (100)	4 (66.7)	33 (67.3)
Race, *n* (%)								
Asian	1 (7.7)	0 (0.0)	0 (0.0)	0 (0.0)	0 (0.0)	0 (0.0)	1 (16.7)	2 (4.1)
Black or African American	1 (7.7)	3 (50.0)	4 (66.7)	6 (100)	3 (50.0)	4 (66.7)	1 (16.7)	22 (44.9)
White	11 (84.6)	3 (50.0)	1 (16.7)	0 (0.0)	3 (50.0)	2 (33.3)	4 (66.7)	24 (49.0)
Other	0 (0.0)	0 (0.0)	1 (16.7)	0 (0.0)	0 (0.0)	0 (0.0)	0 (0.0)	1 (2.0)
Ethnicity, *n* (%)								
Hispanic or Latino	5 (38.5)	1 (16.7)	1 (16.7)	0 (0.0)	1 (16.7)	1 (16.7)	0 (0.0)	9 (18.4)
Not Hispanic or Latino	8 (61.5)	5 (83.3)	5 (83.3)	6 (100.0)	5 (83.3)	5 (83.3)	6 (100.0)	40 (81.6)
BMI, kg/m^2^, mean (SD)	26.1 (3.1)	29.0 (2.1)	26.4 (2.0)	28.3 (2.6)	28.9 (2.4)	27.9 (1.8)	25.8 (3.5)	27.3 (2.8)

BMI, body mass index; SD, standard deviation.

A total of 25 participants with Parkinson’s disease were eligible for inclusion in the MAD study [MEDI1341, *n* = 18 (*n* = 9 for each dose cohort); placebo *n* = 7 total]. Participants were mostly male (72.0%) and white (96.0%), with a mean (SD) age of 63.0 (9.0) years, mean (SD) BMI of 26.7 (4.3) kg/m^2^ and mean (SD) weight of 78.6 (14.7) kg. Most participants were at Hoehn and Yahr stage 2 (*n* = 22), with one participant at stage 1 (1200 mg dose) and two at stage 3 (2000 mg dose). The mean (SD) duration since first Parkinson’s disease diagnosis was 5.9 (5.1) years. Demographics and baseline characteristics were comparable across the cohorts and between those receiving MEDI1341 and placebo (**Table 2**). UPDRS total and motor scores were in a similar range at baseline; however, the placebo group tended to have a longer disease duration and lower levodopa equivalent daily dose than participants receiving MEDI1341 (**Table 2**). There were no statistically significant differences in age, UPDRS total, UPDRS motor, MOCA scores or disease duration between treatment groups. Differences in LEDD were statistically significant between groups (Wilcoxon rank sum test comparing each dose to placebo, *P* < 0.001).

**Table 2:** Baseline demographics and characteristics of participants with Parkinson’s disease in the multiple ascending dose study

	Placebo (*N* = 7)	1200 mg (*N* = 9)	2000 mg (*N* = 9)	Overall (*N* = 25)
Age, years, mean (SD)	63.6 (10.08)	60.9 (8.95)	64.6 (8.80)	63.0 (8.98)
Male sex, *n* (%)	5 (71.4)	7 (77.8)	6 (66.7)	18 (72.0)
Race				
White	6 (85.7)	9 (100.0)	9 (100.0)	24 (96.0)
Black or African American	1 (14.3)	0 (0.0)	0 (0.0)	1 (4.0)
Ethnicity, *n* (%)				
Not Hispanic or Latino	5 (71.4)	6 (66.7)	9 (100.0)	20 (80.0)
Hispanic or Latino	2 (28.6)	3 (33.3)	0 (0.0)	5 (20.0)
BMI, kg/m^2^, mean (SD)	25.7 (4.5)	27.5 (3.4)	26.6 (5.2)	26.7 (4.3)
Hoehn and Yahr Stage, *n* (%)^a^				
1	0 (0.0)	1 (11.1)	0 (0.0)	1 (4.0)
2	7 (100.0)	8 (88.9)	7 (77.8)	22 (88.0)
3	0 (0.0)	0 (0.0)	2 (22.2)	2 (8.0)
Duration since first Parkinson’s disease diagnosis, years, mean (SD)^b^	9.1 (7.7)	5.4 (3.7)	4.0 (2.6)	5.9 (5.1)
Levodopa equivalent dose, median (IQR)	400 (100–970)	700 (388–1183)	600 (425–3275)	600 (400–1050)
Unified Parkinson’s disease Rating Scale, mean (SD)				
Part I (Non-Motor Aspects of Experiences of Daily Living)	4.1 (3.1)	7.7 (3.8)	6.3 (5.9)	−
Part II (Motor Aspects of Experiences of Daily Living)	6.4 (4.3)	11.2 (6.2)	6.2 (5.5)	−
Part III (Motor Examination)	28.3 (15.2)	27.7 (12.7)	20.7 (7.0)	−
Part IV (Motor Complications)	3.6 (2.5)	3.1 (2.4)	3.2 (5.2)	−
Total	42.4 (18.0)	49.7 (14.3)	36.4 (16.9)	−
MoCA score, mean	27.7 (1.5)	27.2 (2.1)	27.8 (1.0)	27.6 (1.5)

^a^Stages: 1 = unilateral involvement only; 2 = Bilateral involvement without impairment of balance; 3 = mild-to-moderate bilateral disease; some postural instability; physically independent.

^b^Duration since first Parkinson’s disease diagnosis was approximated if the full diagnosis date was not provided.

IQR, inter-quartile range; MoCA, Montreal Cognitive Assessment; SD, standard deviation.

### Safety

In the SAD study in healthy participants, MEDI1341 was found to have a favourable safety profile in comparison with placebo and was well tolerated following a single intravenous infusion of 70, 210, 400, 1200, 2400, or 4500 mg. A total of 23 (46.9%) out of the 49 healthy participants reported any TEAE (MEDI1341, *n* = 17; placebo, *n* = 6), all of which were mild or moderate in severity (**Table 3**). The most common TEAEs were headache (16.3%), nausea (10.2%), procedural site reaction (10.2%), and post-lumbar puncture syndrome (8.2%; Supplementary Table 3). The most common system organ classes for TEAEs overall were injury, poisoning, and procedural complications, which were related to administration of MEDI1341 or placebo and accounted for 18 TEAEs in 12 participants (24.5%; Supplementary Table 3). These included procedural site reaction (10.2%), post lumbar puncture syndrome (8.2%), procedural pain (6.1%), post procedural contusion (4.1%), procedural headache (4.1%), post procedural swelling (2.0%) and thermal burn (2.0%). No participant discontinued treatment due to a TEAE. There were notably more TEAEs in participants receiving 1200 mg MEDI1341 compared with the other cohorts. Overall, 16 TEAEs in six participants (12.2%) were considered treatment-related, all of which were mild in severity. The most commonly reported treatment-related TEAE was headache, reported for three (6.1%) participants, one in each of the 400, 1200, and 4500 mg MEDI1341 dose groups (**Table 3**).

**Table 3:** Summary of treatment-emergent and treatment-related treatment-emergent adverse events following single IV dosing of MEDI1341 in healthy participants

	Placebo (*N* = 13)	70 mg (*N* = 6)	210 mg (*N* = 6)	400 mg (*N* = 6)	1200 mg (*N* = 6)	2400 mg (*N* = 6)	4500 mg (*N* = 6)	Overall (*N* = 49)
Participants with TEAEs	6 (46.2)	1 (16.7)	1 (16.7)	4 (66.7)	6 (100.0)	3 (50.0)	2 (33.3)	23 (46.9)
Participants with SAEs	0 (0.0)	0 (0.0)	0 (0.0)	0 (0.0)	0 (0.0)	0 (0.0)	0 (0.0)	0 (0.0)
Participants discontinued due to TEAEs	0 (0.0)	0 (0.0)	0 (0.0)	0 (0.0)	0 (0.0)	0 (0.0)	0 (0.0)	0 (0.0)
Intensity (all TEAEs)								
Mild	6 (46.2)	1 (16.7)	1 (16.7)	4 (66.7)	6 (100)	3 (50.0)	2 (33.3)	23 (46.9)
Moderate	2 (15.4)	0 (0.0)	0 (0.0)	0 (0.0)	1 (16.7)	0 (0.0)	0 (0.0)	3 (6.1)
Participants with treatment-related TEAEs	0 (0.0)	0 (0.0)	0 (0.0)	2 (33.3)	2 (33.3)	0 (0.0)	1 (16.7)	6 (12.2)
Nervous system disorders	0 (0.0)	0 (0.0)	0 (0.0)	1 (16.7)	2 (33.3)	0 (0.0)	1 (16.7)	4 (8.2)
Gastrointestinal disorders	0 (0.0)	0 (0.0)	1 (16.7)	1 (16.7)	0 (0.0)	0 (0.0)	1 (16.7)	3 (6.1)
General disorders and administration site conditions	0 (0.0)	0 (0.0)	0 (0.0)	1 (16.7)	0 (0.0)	0 (0.0)	1 (16.7)	2 (4.1)
Blood and lymphatic system disorders	0 (0.0)	0 (0.0)	0 (0.0)	1 (16.7)	0 (0.0)	0 (0.0)	0 (0.0)	1 (2.0)
Cardiac disorders	0 (0.0)	0 (0.0)	0 (0.0)	1 (16.7)	0 (0.0)	0 (0.0)	0 (0.0)	1 (2.0)

All data are presented as number and percentage of participants unless otherwise indicated. TEAE, treatment-emergent adverse event.

Two SAEs were reported in the SAD study. One participant experienced severe post-lumbar puncture syndrome during screening, which resolved following treatment with ketorolac tromethamine, intravenous fluids, hospital admission and 2 weeks of bed rest. The event was related to the study procedure, and the participant discontinued from the study prior to study drug administration. Cardiac arrhythmia was reported in one participant who had an overdose of MEDI1341 (108.1 mg instead of 70 mg). An echocardiogram performed the day after dosing revealed pre-existing mild left atrial enlargement of unknown cause suspected to be the underlying cause of arrhythmia. The Investigator did not consider the event to be treatment-related. The participant continued to complete safety assessments.

Ophthalmic assessments found no signs of scleral thickening or mononuclear infiltrates in the corneo-scleral junction.

In the SAD study, there were no treatment- or dose-related trends in serum clinical chemistry, haematology, or urinalysis data. Two participants had elevated aspartate aminotransferase and blood creatine phosphokinase, both resolved within 2 weeks and were documented as TEAEs. No treatment- or dose-related trends in supine, standing, or orthostatic systolic or diastolic blood pressure were reported. No treatment- or dose-related trends in ECG parameters were noted following treatment. Physical examinations revealed no clinically significant findings.

In the MAD study, MEDI1341 was found to have a favourable safety profile in comparison with placebo in participants with Parkinson’s disease and was well tolerated following three repeat intravenous infusions of 1200 mg or 2000 mg administered every 4 weeks. A total of 10 (40.0%) out of the 25 participants with Parkinson’s disease reported any TEAE (MEDI1341, *n* = 9; placebo, *n* = 1), of which most (23/27) were mild in severity (**Table 4**). The most frequently reported TEAEs were fall and nausea (*n* = 2 [8.0%] each). The most common system organ class for TEAEs overall was gastrointestinal disorders, which were reported for four (16.0%) participants and included nausea, diarrhoea, and large intestine polyps (Supplementary Table 4). One participant receiving 1200 mg MEDI1341 reported four treatment-related TEAEs (fatigue, headache, nausea and rash). All treatment-related TEAEs were mild with the exception of fatigue which was moderate in severity (**Table 4**). No treatment-related TEAEs were reported in participants receiving MEDI1341 2000 mg or placebo. No SAEs were reported during the study. No participant discontinued treatment due to a TEAE. Ophthalmic assessments found no signs of scleral thickening or mononuclear infiltrates in the corneo-scleral junction.

**Table 4:** Summary of treatment-emergent and treatment-related treatment-emergent adverse events following multiple IV doses of MEDI1341 in participants with Parkinson’s disease

	Placebo (*N* = 7)	1200 mg (*N* = 9)	2000 mg (*N* = 9)	Overall (*N* = 25)
Participants with TEAEs [number of TEAEs]	1 (14.4) [3]	4 (44.4) [16]	5 (55.6) [8]	10 (40.0) [27]
Participants with SAEs	0 (0.0)	0 (0.0)	0 (0.0)	0 (0.0)
Participants discontinued due to TEAEs	0 (0.0)	0 (0.0)	0 (0.0)	0 (0.0)
Intensity (all TEAEs)				
Mild	1 (14.3)	2 (22.2)	5 (55.6)	7 (28.0)
Moderate	1 (14.3)	2 (22.2)	0 (0.0)	3 (12.0)
Participants with treatment-related TEAEs				
Overall	0 (0.0)	1 (11.1)	0 (0.0)	1 (4.0)
Fatigue	0 (0.0)	1 (11.1)	0 (0.0)	1 (4.0)
Headache	0 (0.0)	1 (11.1)	0 (0.0)	1 (4.0)
Nausea	0 (0.0)	1 (11.1)	0 (0.0)	1 (4.0)
Rash	0 (0.0)	1 (11.1)	0 (0.0)	1 (4.0)

All data are presented as number and percentage of participants unless otherwise indicated.

TEAE, treatment-emergent adverse event.

There were no treatment- or dose-related trends in serum clinical chemistry, haematology, or urinalysis data and no clinical laboratory results met the criteria for AE reporting in the MAD study. No treatment- or dose-related trends for supine, standing, or orthostatic systolic or diastolic blood pressure were reported. No treatment- or dose-related trends in ECG parameters were noted following treatment. Physical examinations revealed no clinically significant findings, and there were no appreciable trends for changes in the neurological examinations during the course of the study.

### Pharmacokinetics

**Figure 2:**
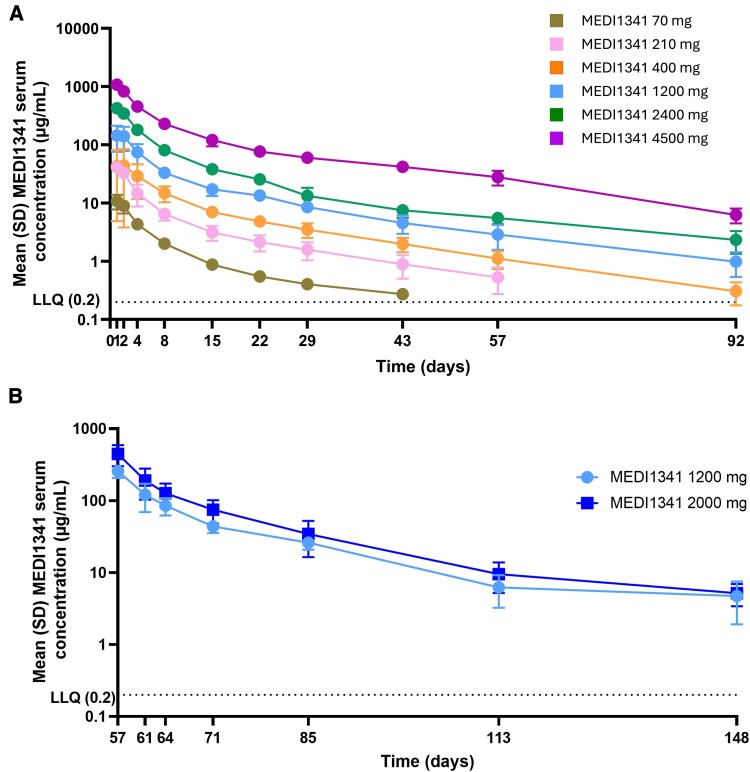
Serum concentrations of MEDI1341 following (**A**) single IV dose of MEDI1341 in healthy participants and (**B**) multiple IV doses after third infusion (Day 57) in participants with Parkinson’s disease. Individual data points are shown in Supplementary Fig. 1. IV, intravenous; LLQ, lower limit of quantitation; SD, standard deviation. *N* = 6 for each dose cohort and *N* = 13 for placebo in the SAD. *N* = 9 for each dose cohort and *N* = 7 for placebo in the MAD.

In the SAD study, increases in C_max_ were dose proportional in healthy participants over the dose range 70–4500 mg MEDI1341 **(**Supplementary Table 5). Increases in AUC _0–∞_ and AUC_0–t_ were dose proportional over the dose range 70–2400 mg and supra-proportional from 2400 to 4500 mg, with a nearly 3-fold increase in geometric mean AUC_0–∞_ and AUC_0–t_ for the 1.88-fold increase in dose (Supplementary Tables 5 and 6). Occurrence of median t_max_ was variable but generally observed 1 hour (i.e. around the end of the infusion) after a single intravenous administration of MEDI1341 [median (range) 0.983 (0.92–9.00), 0.975 (0.93–9.02) and 0.983 (0.98–9.00) hours for 70, 210, and 2400 mg doses, respectively]. At the 400, 1200 and 4500 mg dose levels, respective median (range) t_max_ were 17.0 (0.93–67.33), 9.06 (0.97–72.92), and 5.01 (0.98–9.00) hours after the start of infusion. Following a single intravenous administration, serum concentrations of MEDI1341 declined after C_max_ in a biphasic manner (Fig. 2A and Supplementary Fig. 1A) with geometric mean t_1/2λz_ ranging from 16.6 to 24.3 days across doses. Geometric mean CL, *V*_z_ and *V*_ss_ were similar across the dose range 70 to 2400 mg (ranging from 0.927 to 1.09 L/day, 22.6 to 32.6 L, and 13.6 to 18.5 L, respectively) and lower at the 4500 mg dose level (0.590 L/day, 16.0 L, and 11.1 L, respectively). Across the dose levels, geometric mean MEDI1341 CSF concentrations were highest on Day 8 compared with Days 15 and 29 (Supplementary Table 7). For all dose levels and time points, individual CSF concentrations of MEDI1341 were <1% of their respective serum concentrations and the geometric mean concentration ratios appeared to be similar or slightly decreasing with increasing dose. Additional pharmacokinetic data are presented in Supplementary Table 8.

In the MAD study in participants with Parkinson’s disease, increases in C_max_ and AUC_0-τ_ were dose proportional between 1200 mg and 2000 mg MEDI1341 doses (Supplementary Table 9). Median t_max_ occurred approximately 1 hour after the initiation of intravenous administration of MEDI1341 [median (range) 1.08 (0.967–25.4) and 1.05 (0.934–50.4) hours after the first dose (Supplementary Table 10)] and median (range) 1.06 (1.00–25.1) and 1.04 (0.917–9.07) after the third dose (Supplementary Table 11) for the 1200 and 2000 mg dose levels, respectively]. Following intravenous administration, serum concentrations of MEDI1341 declined after C_max_ in a biphasic manner (Fig. 2B and Supplementary Fig. 1B) with geometric mean *t*_1/2λz_ of 18.2 days following the last 1200 mg dose and 19.5 days following the last 2000 mg dose. Geometric mean CL and V_z_ following the last dose were comparable between the 1200 (0.636 L/day and 16.4 L, respectively) and 2000 mg (0.669 L/day and 19.2 L, respectively) doses. Geometric mean CSF MEDI1341 concentrations were higher on Day 61 than on Day 85 for both doses (Supplementary Table 10). For both doses at all time points, individual CSF concentrations of MEDI1341 were <1% of their respective serum concentrations, and the geometric mean concentration ratios appeared to be similar between doses. Additional pharmacokinetic data are presented in Supplementary Tables 11 and 12.

### Pharmacodynamics

**Figure 3:**
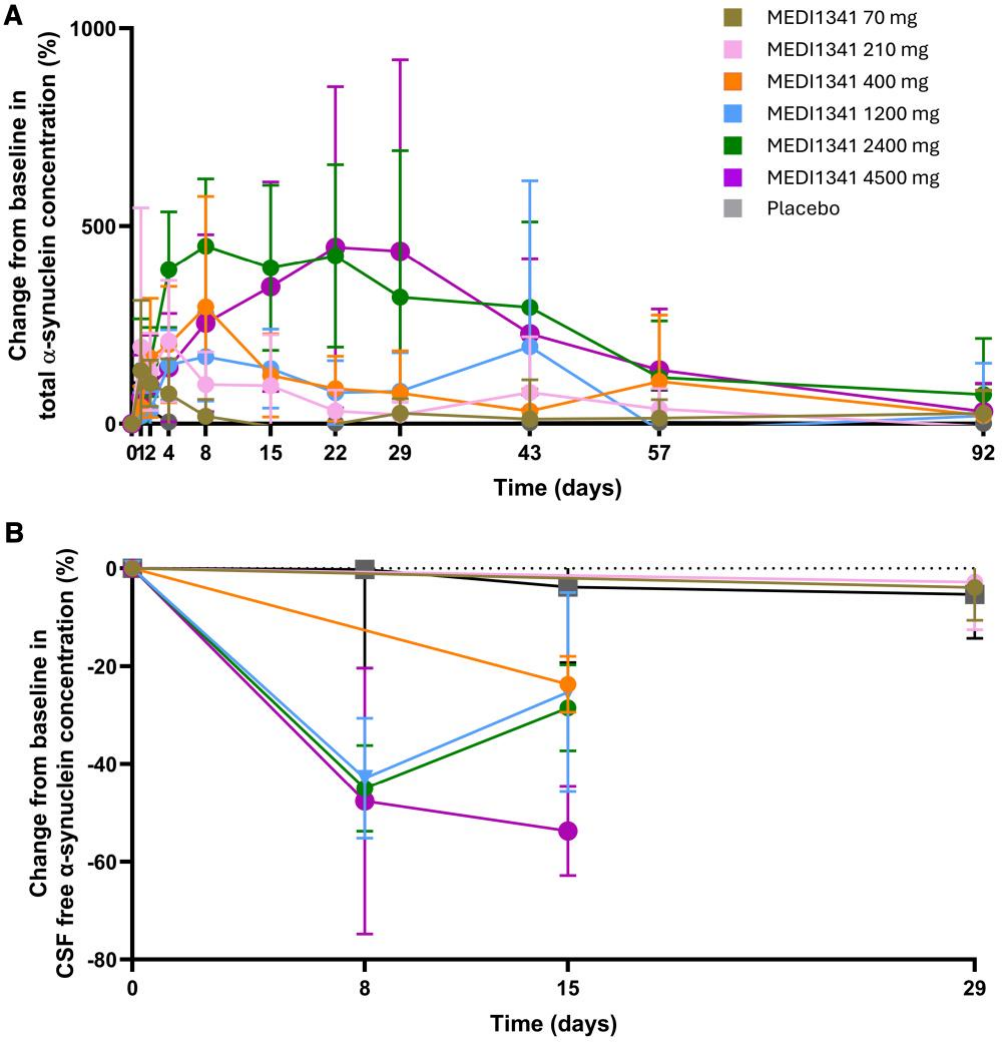
Mean (SD) change from baseline in (**A**) total α-synuclein concentration in plasma and (**B**) CSF-free α-synuclein concentration following a single MEDI1341 IV dose in healthy participants. Individual data points are shown in Supplementary Fig. 2. CSF, cerebrospinal fluid; IV, intravenous; SD, standard deviation. *N* = 6 for each dose cohort and *N* = 13 for placebo in the SAD. *N* = 9 for each dose cohort and *N* = 7 for placebo in the MAD.

In the SAD study, following a single intravenous infusion of 70–4500 mg MEDI1341 in healthy participants, mean change from baseline total α-synuclein concentration in plasma increased in a dose-dependent manner before decreasing back to baseline values (Fig. 3A). Between-participant variability was high (Supplementary Fig. 2A). For participants receiving doses of 70, 210, 400, 1200, 2400 or 4500 mg, the greatest mean (SD) percentage changes from baseline in plasma total α-synuclein were observed on Day 1 [134.3% (177.76)], Day 4 [209.1% (153.65)], Day 8 [295.5% (279.58)], Day 43 [195.1% (419.55)], Day 8 [449.5% (169.96)] and Day 22 [446.8% (406.28)], respectively. The levels of total α-synuclein in plasma began to drop from Days 8, 15, 22, 43, 57 and 92 for the 70, 210, 400, 1200, 2400 and 4500 mg doses, respectively. No obvious trend was observed for the placebo group. In healthy participants, free α-synuclein concentrations in CSF decreased in a dose-dependent manner. Suppression was greatest at the 4500 mg dose, with mean (SD) percentage changes from baseline of −47.6% (27.2) and −53.7% (9.15) on Day 8 and Day 15, respectively (Fig. 3B), and median (range) percentage changes from baseline of −58.3% (−62.3, 7.8) and −53.6% (−64.5, −43.3) on Day 8 and Day 15, respectively (Supplementary Table 13). Individual data for Fig. 3B are shown in Supplementary Fig. 2B. Exploratory *post hoc* statistical testing using Welch’s *t*-tests showed a statistically significant effect compared with placebo at doses of 400 mg (*P* = 0.01), 1200 mg (*P* = 0.01), 2400 mg (*P* = 0.003), 4500 mg (*P* < 0.001).

**Figure 4:**
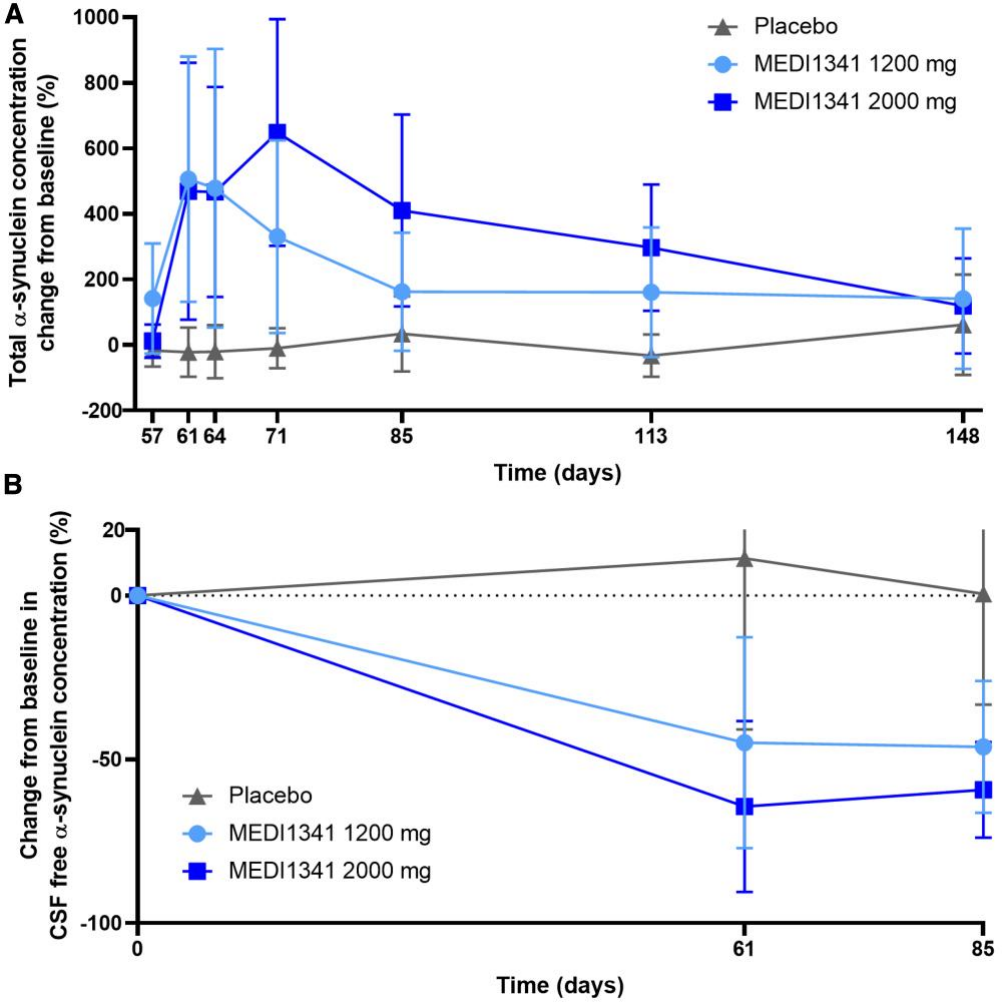
Mean (SD) change from baseline following multiple MEDI1341 IV doses in participants with Parkinson’s disease in (**A**) total α-synuclein concentration in plasma [following the last administered dose (Day 57)] and (**B**) CSF-free α-synuclein concentration. Individual data points are shown in Supplementary Fig. 3. CSF, cerebrospinal fluid; IV, intravenous; SD, standard deviation. *N* = 6 for each dose cohort and *N* = 13 for placebo in the SAD. *N* = 9 for each dose cohort and *N* = 7 for placebo in the MAD.

In the MAD study, following the third intravenous infusion of MEDI1341 (Day 57) in participants with Parkinson’s disease, mean (SD) change from baseline in plasma total α-synuclein increased and peaked at Days 61 (506% [374]) and 71 (648% [346]) for the 1200 and 2000 mg doses, respectively, before decreasing towards baseline levels (Fig. 4A). Between-participant variability was high (Supplementary Fig. 3A). No obvious trend was observed with placebo. Following administration of the third intravenous infusion of MEDI1341 in participants with Parkinson’s disease, free α-synuclein concentration in CSF was significantly lower compared with placebo and decreased in a dose-dependent manner. Mean (SD) percentage changes from baseline for free α-synuclein in CSF were −‍44.9% (32.2), −64.4% (26.1), and 11.3% (52.2) for MEDI1341 1200 mg, MEDI1341 2000 mg and placebo, respectively, at Day 61, and −46.2% (20.1), −59.3% (14.6), and 0.457% (33.8), respectively, at Day 85 (Fig. 4B). Individual data for Fig. 4B are shown in Supplementary Fig. 3B. Median (range) percentage changes from baseline for free α-synuclein in CSF were −55.5% (−77.3, 7.38), −75.2% (−81.9, −13.0), and −7.09% (−27.4, 127) for MEDI1341 1200 mg, MEDI1341 2000 mg, and placebo, respectively, on Day 61, and −51.1% (−66.9, −17.3), −59.0% (−80.3, −37.6), and 12.6% (−36.5, 35.8), respectively, on Day 85 (Supplementary Table 14). Exploratory *post hoc* statistical testing using Welch’s *t*-tests showed a statistically significant effect compared with placebo at doses of 1200 mg (*P* = 0.009) and 2000 mg (*P* = 0.001).

### Immunogenicity

In the SAD study, overall, three healthy participants (MEDI1341, *n* = 1; placebo, *n* = 2) were ADA-positive pre-dose. The emergence of ADAs in healthy participants was generally observed at later timepoints post-infusion. Of the participants receiving MEDI1341 or placebo, 11 (30.6%) and three (25.0%), respectively, were ADA positive, and one (2.0%) and zero (0.0%), respectively, were borderline ADA positive. The borderline positive ADA result was reported on Day 92 in a participant receiving 70 mg MEDI1341, with their other assessments reported as ADA negative. The earliest post-dose ADA-positive result was reported on Day 57. Across dose groups, the proportion of participants with positive ADA results ranged from 0.0% (4500 mg dose) to 50.0% (210 and 1200 mg doses), with no apparent dose-related trend. Across all timepoints, titres ranged from 50 to 12100 in participants receiving MEDI1341 and from 50 to 1350 in participants receiving placebo. There was no evidence to indicate that ADA presence had an impact on pharmacokinetics, pharmacodynamics, or safety. However, MEDI1341 serum concentrations declined faster during the terminal phase of the pharmacokinetic profile of the participant with a titre of 12100 compared with the other participants in the same dose group (1200 mg).

In the MAD study, no ADAs were observed pre-dose in participants with Parkinson’s disease. Overall, two (22.2%) participants receiving multiple 1200 mg infusions of MEDI1341 developed treatment-emergent ADAs with titres ranging from 50 to 150. ADAs were not observed in the participants receiving 2000 mg MEDI1341 or placebo. Presence of ADAs had no impact on pharmacokinetics, pharmacodynamics, or safety.

### Clinical manifestations

For participants with Parkinson’s disease, no obvious changes were noted in cognition, based on MoCA (Supplementary Table 15). There were minimal fluctuations in the total and individual parts of the MDS–UPDRS score, without any obvious trends for changes from baseline or notable differences between treatment groups (Supplementary Table 16).

## Discussion

MEDI1341 is the first monoclonal antibody targeted against α-synuclein to demonstrate a > 50% reduction in free α-synuclein in CSF of healthy participants and participants with Parkinson’s disease. Reduction of free α-synuclein in CSF was generally observed to be dose-dependent. Across all dose levels and time points, individual participant CSF MEDI1341 concentrations were <1% of their respective MEDI1341 serum concentrations, as expected for an IgG1 monoclonal antibody. The relationship between reductions in free α-synuclein in CSF and clinical effect remains to be studied, as this requires a longer period of follow-up in a larger study. This is currently being investigated in patients with multiple system atrophy, another synucleinopathy, in an on-going Phase 2 trial of MEDI1341 (ClinTrials.gov ID: NCT05526391), which aims to evaluate whether a reduction in free α-synuclein in human CSF translates into slowing of disease progression. The efficacy of monoclonal antibodies in synucleinopathies is likely to vary with disease stage. Given the underlying pathology of Parkinson’s disease, removal of α-synuclein early in the disease course, prior to involvement of other pathways which may independently contribute to disease progression (e.g. inflammation), may offer greater therapeutic benefit. However, the level of α-synuclein clearance that must be achieved to provide meaningful clinical outcomes remains unclear. Additionally, the relationship between indirect measures of target engagement (e.g. CSF-free α-synuclein measurements) and pathology in the brain parenchyma is not fully understood. The development of α-synuclein PET ligands represents a promising avenue to further clarify the relationship between fluid biomarkers and parenchymal pathology, which will advance our understanding of the clinical effects of α-synuclein targeting therapies.

There are currently four other monoclonal antibodies targeted towards α-synuclein that have entered clinical development. Cinpanemab, a human-derived IgG1 monoclonal antibody, binds preferentially to aggregated forms of extracellular α-synuclein and does not target monomeric forms of α-synuclein.^28,29,33^ A phase 2 trial in patients with Parkinson’s disease found cinpanemab had no significant effect on clinical disease progression compared with placebo.^28^ Prasinezumab, a humanized IgG1 monoclonal antibody that also selectively targets aggregated α-synuclein rather than monomeric α-synuclein, showed no overall impact on the progression of Parkinson’s disease,^25-27,34^ and reported no statistically significant changes in free α-synuclein in the CSF compared with placebo in a phase 1b study (quantitative results were not reported).^34^ Target engagement measures were not included in the subsequent phase 2 studies due to a lack of well-developed tests.^25^ Data on α-synuclein depletion in human CSF with ABBV-0805 have not been reported, and its development has been discontinued.^35,36^ LU AF82422, an anti-α-synuclein human IgG1 monoclonal antibody, which binds all known forms of aggregated α-synuclein, has been shown to reduce α-synuclein by ∼40% in human CSF at a dose of 9.0 g.^37,38^ This effect observed with LU AF82422 was of apparent similar magnitude to that observed with MEDI1341 in our study, but required significantly higher doses, which may be challenging for longer-term development. It is also not clear whether the onward development of LU AF82422 is evaluating those higher doses.

Methodological differences when assessing free α-synuclein concentrations in the CSF make comparison between α-synuclein targeting monoclonal antibodies difficult, and assays may be influenced by the presence of drugs. The assay used in our analysis included an initial immunoprecipitation step to deplete all MEDI1341–α-synuclein complexes and the remaining ‘free’ α-synuclein was measured using an immunoassay. In the analysis described in the LU AF82422 programme, Lundbeck reported using a proprietary antibody as the capture antibody, presuming that the presence of a bound antibody would not interfere in the assay due to occupied binding sites.^37^ Comparisons are further complicated by monoclonal specificity, for example, MEDI1341 binds all forms of α-synuclein, whereas LU AF82422 is reported to bind aggregated forms only.^39^ Newer seed amplification assays also provide opportunities to assess the impact of α-synuclein targeting therapies on fluorescence-time curves.^40^ However, the approach is limited in being semi-quantitative, which makes it helpful for diagnosis, potentially distinguishing between Parkinson’s disease and multiple system atrophy, and possibly to demonstrate binding to α-synuclein aggregates, but not as a measure of therapeutic effectiveness. It is not yet clear what a readout of disease modification would look like in this assay.

MEDI1341 was found to have a favourable safety profile and was generally well tolerated across all doses in both healthy participants and participants with Parkinson’s disease. Common TEAEs included headache, nausea, procedural site reactions and post-lumbar puncture syndrome. All treatment-related TEAEs were mild or moderate in severity. Two SAEs occurred during the SAD study but were not considered treatment-related. Whilst TEAEs occurred more frequently in participants with Parkinson’s disease receiving MEDI1341 compared with those receiving placebo in the MAD study, clinical laboratory evaluations, ECG data, physical, injection site and infusion reaction assessments, as well as ophthalmic assessments did not reveal any trends that would suggest that MEDI1341 is associated with an adverse safety profile. Moreover, MoCA and MDS–UPDRS assessments were all within the expected range, indicating that clinical features of Parkinson’s disease remained relatively stable during the study and were unaffected by treatment assignment. A larger sample size and treatment duration will further inform the safety profile.^31^

Increases in C_max_ were dose proportional following intravenous administration of MEDI1341. Increases in AUC_0–∞_ and AUC_0-t_ were dose proportional over the dose range 70 to 2400 mg but supra-proportional between 2400 and 4500 mg in healthy participants in the SAD study. The supra-proportional increase in AUCs at the highest dose investigated coincided with saturation of α-synuclein in plasma, as shown by the absence of further increase in total α-synuclein plasma concentration at 4500 mg. As the pharmacokinetic assay measured free MEDI1341 concentration, it is hypothesized that after the α-synuclein target (which is in the ng range) has been saturated in plasma, a greater concentration of free MEDI1341 becomes available in the systemic circulation. Of note, geometric mean CL and Vz were broadly similar between participants with Parkinson’s disease receiving multiple MEDI1341 doses and healthy participants after a single 4500 mg dose. Individual MEDI1341 t_max_ were variable, but generally observed at the end of the 1-hour infusion. MEDI1341 geometric mean CL was higher than that reported for cinpanemab and LU AF82422 but similar to prasinezumab. The *V*_ss_ observed for MEDI1341 was higher than for cinpanemab, lower than prasinezumab but similar to LU AF82422, all of which are monoclonal antibodies targeting α-synuclein.^33,34,37,41^ The relatively long MEDI1341 t_1/2λz_ of 16.6–24.3 days, typical for an IgG1 monoclonal antibody, supports the 4-weekly dosing regimen currently being tested in the on-going Phase 2 trial.

ADA titres were generally low and ranged from 50 to 12100 in the SAD study and from 50 to 150 in the MAD study. As the MEDI1341 dose increased, there did not appear to be an increased number of participants with borderline positive and/or positive ADA results, and there was no discernible trend in titre values. Positive ADA results did not appear to impact the safety, pharmacokinetics or pharmacodynamics of MEDI1341.

There was a statistically significant difference in the levodopa equivalent daily dose between treatment groups, with the patients on placebo having a lower overall levodopa equivalent daily dose. However, variations in levodopa equivalent daily dose are unlikely to have influenced changes in CSF-free α-synuclein concentrations, as these were assessed based on change from baseline within each dose groups (pre- and post-treatment comparison).

This study is limited by the relatively small sample size, which may affect the generalizability of the findings to a broader population and offers only a preliminary assessment of safety and immunogenicity.^42^ Moreover, long-term outcomes cannot be assessed due to the relatively short follow-up period. The safety and immunogenicity of MEDI1341 will be further characterized over an extended period during the on-going phase 2 study.

In summary, the first in-human studies of single and multiple doses of MEDI1341 found it was generally well-tolerated and showed a similar safety profile to placebo in healthy participants (up to 4500 mg) and participants with Parkinson’s disease (up to 2000 mg). Increases in AUCs were dose-proportional up to 2400 mg in healthy participants and 2000 mg in participants with Parkinson’s disease. Notably, administration of MEDI1341 resulted in a > 50% reduction of free α-synuclein in CSF. The relationship between clinical effect and the reductions in free α-synuclein in CSF and the demonstrated reduced spread of relevant pathology in non-clinical species remains to be shown. The clinical outcomes for participants with multiple system atrophy following treatment with MEDI1341 are being evaluated in an on-going proof-of-concept Phase 2 trial.

## Supplementary Material

fcaf304_Supplementary_Data

## Data Availability

Data underlying the findings described in this manuscript may be obtained in accordance with AstraZeneca’s data sharing policy described at https://astrazenecagrouptrials.pharmacm.com/ST/Submission/Disclosure. The data that support the findings of these studies may be obtained from the CPRD (https://cprd.com/) but restrictions may apply to the availability of these data, which were used under license for the current studies.
