# Supplementary material for: Phase 1, randomized trials of MEDI1341: cerebrospinal fluid free α-synuclein lowered by >50%
Source: Brain Commun. 2025 Aug 19;7(5):fcaf304. doi: 10.1093/braincomms/fcaf304 (PMC12421129; doi:10.1093/braincomms/fcaf304)
Supplement: fcaf304_Supplementary_Data [file fcaf304_supplementary_data.docx]

**Supplementary material**

**SAD study inclusion criteria**

For inclusion in the study participants should fulfil the following criteria:

1. Healthy men and women of non-childbearing potential (i.e., postmenopausal or surgically sterile), aged 18 to 65 (changed during the study via protocol amendment from 80- years) years inclusive, at screening could be enrolled. For women, postmenopausal status was confirmed by serum follicle-stimulating hormone (FSH) testing at screening.
2. Postmenopausal women must have had ≥12 months of spontaneous amenorrhea and must have had a negative serum or urine pregnancy test result at screening or check-in. Surgically sterile women were defined as those who have had a hysterectomy, bilateral ovariectomy (oophorectomy), bilateral salpingectomy, or bilateral tubal ligation. Women who were surgically sterile had to provide documentation of the procedure by an operative report or relevant medical records, or by ultrasound, and must have had a negative serum or urine pregnancy test at screening or check-in.
3. Men who were biologically capable of fathering children must have agreed and committed to use of an adequate form of double-barrier contraception and refrain from sperm donation for the duration of the treatment period and for 5 half-lives plus 14 days after the last administration of study drug. A male participant was considered capable of fathering children even if his sexual partner was sterile or using contraceptives.
4. Weight ≥50 kg and BMI between 18.0 and 32.0 kg/m^2^, inclusive, at screening and at check-in.
5. Participants understood the nature of the study and provided signed and dated written informed consent prior to initiation of any study-related procedures.
6. Participants were healthy, in the opinion of the Investigator, with no clinically significant abnormality identified on the medical or laboratory evaluation at screening. A participant with a clinical abnormality or laboratory test result(s) outside the reference range for his/her age group was included only if the Investigator considers that the finding did not introduce additional risk factors and did not interfere with the study procedures.
7. Participants must have had a 12-lead electrocardiogram (ECG) recorded at screening that, in the opinion of the Investigator, was normal for the appropriate age group and showed no abnormalities that would compromise safety in this study.
8. Participants must have had no clinically significant findings on the clinical neurological examinations at screening and at baseline (Day -1) or on the ophthalmic examination at screening.
9. Participants understood and complied with protocol requirements, instructions, and protocol-stated restrictions.
10. Participants agreed not to post any personal medical data related to the study or information related to the study on any website or social media site (e.g., Facebook, Twitter, etc.) until the study had been completed.

**SAD study exclusion criteria**

Participants should not enter the study if any of the following exclusion criteria are fulfilled:

*General*

1. Requires treatment with another biological therapeutic agent.
2. Participation in another study investigating active or passive immunization against α-synuclein, or immunoglobulin G therapy, within 6 months before screening and until after the final follow-up visit.
3. Use of any experimental medication, device, or biologic within 3 months or 5 half-lives of that investigational product (IP; whichever is longer) prior to screening and until after the final follow-up visit.
4. Donation of blood or plasma within 2 months prior to screening and until 2 months after the final follow-up visit.
5. Poor venous access, such that intravenous (IV) drug delivery or pharmacokinetic/safety blood sampling would be difficult.
6. Nicotine use within 6 months before screening and until the final follow-up visit (a past history of nicotine use prior to this period was not exclusionary.).
7. Inability to abstain from strenuous physical activity from 72 hours prior to admission to the clinical research unit (CRU) until after the final follow-up visit.
8. Inability to comply with study-related restrictions and requirements related to consumption of caffeine and energy drinks, alcohol, provision of meals and snacks, activity, blood donation, and contraception.
9. Current employment by AstraZeneca or by a contract research organization or clinical study site participating in this study, or a first-degree relative of an AstraZeneca employee or of an employee at a participating contract research organization or clinical study site.

*Medical history*

1. History of acute neuronal injury (central or peripheral) within the previous 12 months; history of significant head injury or neurotrauma associated with prolonged loss of consciousness within the participant’s lifetime; history of peripheral neuropathy or neuritis, neurodegenerative disorders, or other neurological diseases, including seizures (except childhood febrile convulsions), or serious infectious diseases affecting the brain, including conditions that are known or hypothesized to be associated with disruption in the blood-brain barrier.
2. Considered to be at a high risk of developing a stroke, including a history of carotid artery disease or surgery, transient ischemic attacks, reversible ischemic neurological deficits; or other abnormalities of the brain vessels, including but not limited to, cerebral artery aneurysms or arteriovenous malformations; or a history of paroxysmal atrial fibrillation or atrial flutter
3. Significant medical history of dizziness, blackouts, fainting, or vaso-vagal attacks.
4. History of any significant ophthalmic disorder, including (but not necessarily limited to) congenital, genetic or acquired [local or systemic] conditions affecting the retina or choroid. Mature cataracts (Grade ≥4 per the Lens Opacities Classification System III) or lower grade cataracts that are otherwise considered by the examining ophthalmologist to prevent adequate examination of the uveal tract or posterior ocular segment are considered exclusionary. However, an age-related cataract with age-appropriate lens opacification and no other important identified secondary causes (e.g., diabetes, corticosteroids, vitamin deficiencies, trauma, radiation, or systemic fluid and electrolyte disturbances) is not necessarily exclusionary.
5. History of severe allergy/hypersensitivity reactions or ongoing allergy/hypersensitivity reactions, or history of hypersensitivity to immunizations or immunoglobulins.
6. History of any significant psychiatric disorder according to the criteria of the Diagnostic and Statistical Manual of Mental disorders, 5th Edition (APA 2013), which, in the opinion of the Investigator, could be detrimental to participant safety or could compromise study data interpretation.
7. History of alcohol abuse, defined as an average weekly intake of >21 units or an average daily intake of >3 units for men and an average weekly intake of >14 units or an average daily intake of >2 units for women. One unit is equivalent to a half pint (250 mL) of beer, 1 measure (25 mL) of spirits, or 1 glass (125 mL) of wine.
8. History of cancer within 5 years of screening, or between screening and randomization, with the exception of non-metastatic basal cell carcinoma of the skin, carcinoma in situ of the cervix, or non-progressive prostate cancer.
9. History of drug abuse or positive test for drugs of abuse or alcohol at screening or at baseline on Day -1.

*Concomitant medications, procedural contraindications, and contraception*

1. Use of prescription or non-prescription drugs, including vitamins and herbal and dietary supplements, within 7 days or 5 half-lives (whichever is longer) prior to the administration of study treatment. This was applicable unless, in the opinion of the Investigator and the Sponsor, the medication did not interfere with the study procedures or compromise participant safety. Routine vaccination within 2 weeks of planned dosing was exclusionary. Medications known to significantly influence coagulation were prohibited.
2. Any contraindication to lumbar puncture, including but not limited to thrombocytopenia or other coagulation disorders (including participants receiving low-dose aspirin, coumarin or other anticoagulants, or low-molecular-weight heparin), the presence of cutaneous or soft-tissue infection overlying or adjacent to the site of lumbar puncture, previous spinal surgery that could complicate access to the subarachnoid space, or conditions associated with raised intracranial pressure, such as a closed head injury within 3 months, or benign intracranial hypertension.

*Physical examinations, electrocardiograms, vital signs, and laboratory tests*

1. Any clinically important abnormality as determined by the Investigator at screening, or between screening and randomization, in physical, neurological, or ophthalmic examination; vital signs; ECG; or clinical laboratory test results that could be detrimental to participant safety or could compromise the study. See exclusion criterion 13 for detailed criteria for the exclusion of participants with cataracts.
2. Any clinically significant abnormality in resting vital signs at screening or Day -1, defined in terms of a supine systolic blood pressure which is outside the range of 90 to 140 mmHg; diastolic blood pressure is outside the range of 50 to 90 mmHg or heart rate (HR) is outside the range 45 to 100 beats per minute (bpm).
3. Any clinically significant abnormality in ECG rhythm, conduction or morphology, including (a) clinically significant PR (PQ) interval prolongation (PR >220 msec); (b) intermittent second or third degree atrioventricular (AV) block (AV block II Mobitz Type 1, Wenkebach, while asleep or at deep rest was not exclusionary); or (c) incomplete, full or intermittent bundle branch block (QRS <115 msec with normal QRS and T wave morphology is acceptable if there was no evidence of left ventricular hypertrophy).
4. Prolonged QTcF >450 msec or family history of long QT syndrome; or shortened QTcF <360 msec, or family history of short QT syndrome at screening or check-in.
5. Positive serologic findings at screening for human immunodeficiency virus (HIV) antibodies, hepatitis B surface antigen, or hepatitis C virus antibodies.
6. For female participants, a positive serum or urine pregnancy test result at screening or check-in.

**MAD study inclusion criteria**

*Age*

1. Participants aged between 40 to 85 years (inclusive) on the day of randomization.

*Type of Participant and Disease Characteristics*

1. Met criteria for a diagnosis of mild-to-moderate idiopathic Parkinson’s disease according to the United Kingdom Parkinson’s disease Society Brain Bank criteria
2. Parkinson’s disease was at stages 1 to 3 using the Hoehn and Yahr scale as modified.
3. Participants receiving medications for idiopathic Parkinson’s disease were on a stable dosing regimen of their medication(s) for ≥1 month before randomization (and their ongoing medications or dosing regimen were not changed for the duration of the study, barring unforeseen circumstances). For participants who were not currently receiving medications to treat idiopathic Parkinson’s disease, there was no expectation of a need to initiate the medications for the duration of the study

*Weight*

1. Participants had a body weight of 45 to 120 kg (inclusive), and a BMI of 18 to 34 kg/m^2^ (inclusive) at screening and check-in for the first infusion.

*Sex*

1. Participants were male or female. Female participants were of non-childbearing potential (postmenopausal and/or surgically sterile).

*Reproduction*

1. Postmenopausal women had ≥12 months of spontaneous amenorrhea and must have had a negative serum pregnancy test result at screening. Surgically sterile women were defined as those who had a hysterectomy, bilateral ovariectomy (oophorectomy), salpingectomy, or bilateral tubal ligation. Women who were surgically sterile were required to provide documentation of the procedure by an operative report, ultrasound, or other verifiable medical documentation.
2. Men who were biologically capable of fathering children agreed and committed to use an adequate form of double-barrier contraception for the duration of the treatment period and for 5 half-lives (100 days) after the last administration of study intervention. A male participant was considered capable of fathering children even if his sexual partner was sterile or on contraceptives.
3. Men who were biologically capable of fathering children also agreed to refrain from sperm donation for the duration of the treatment period and for 5 half-lives or 90 days (whichever was longer) after the last administration of study intervention.

*Informed Consent*

1. Participants in the investigator’s opinion understood the nature of the study and provided signed and dated written informed consent before the conduct of any study-related procedures.

*Other Inclusion Criteria*

1. Participants had to, in the opinion of the investigator, be able to participate in all scheduled evaluations, likely to be compliant, and likely to complete all required tests, including magnetic resonance imaging (MRI) brain scans and lumbar punctures. Note: The investigator assessed the physical and functional needs of the participant at screening, as participation in the study was contingent on the availability and willingness of a caregiver to attend with the participant at all study visits.
2. Participants were able to read, write, and speak fluently in English and/or Spanish.
3. Participants agreed to not post any personal medical data related to the study or information related to the study on any website or social media site (e.g., Facebook, Twitter, Instagram) until the study has been completed.
4. Participants had a Montreal Cognitive Assessment total score of ≥24.

**MAD study exclusion criteria**

*Medical Conditions*

1. In the opinion of the investigator, a recent clinically significant illness (other than Parkinson’s disease), infection, medical/surgical procedure, or significant trauma that occurred within 30 days prior to screening, or between screening and randomization, that was likely to deteriorate, compromise the participant’s safety or ability to complete the study, or compromise the interpretation of the study results (Note: a history of coronavirus disease 2019 [COVID-19] infection with unresolved medical sequelae was considered exclusionary).

2. Presence of a serious or unstable clinically significant illness, including hepatic, renal, gastroenterological, respiratory, cardiovascular, endocrinologic, immunologic or autoimmune disease (e.g., multiple sclerosis), hematologic, or other major diseases, which, in the judgment of the investigator, was poorly controlled or otherwise likely to deteriorate, compromise the participant’s safety, ability to complete the study, or compromise the interpretation of the study results.

3. Significant neurological disease affecting the central nervous system (other than Parkinson’s disease) that, in the opinion of the investigator, may have affected motor function or the ability to complete the study, including but not limited to progressive supranuclear palsy, multiple system atrophy (including MSA-P and MSA-C or other multiple system atrophy terminology: striatonigral degeneration, olivopontocerebellar atrophy, or autonomic failure), postencephalitic parkinsonism, metabolic diseases with parkinsonian signs and symptoms (e.g., Wilson disease, manganese exposure) or other secondary forms of Parkinsonism, and ischemic or traumatic brain injury (including multiple episodes of head trauma, or head trauma resulting in protracted loss of consciousness within the 5 years prior to screening or between screening and randomization).

4. Brain MRI scan that showed clinically significant evidence of malignant, ischemic, demyelinating, structural, or degenerative brain disease or had findings that compromised the safety of lumbar puncture.

5. Had undergone surgery for the treatment of Parkinson’s disease (e.g., pallidotomy, deep brain stimulation, foetal tissue transplantation) or had undergone any other brain surgery at any time, even for non- Parkinson’s disease conditions.

6. Had history of epilepsy or seizures, except febrile childhood seizures.

7. Had history of transient ischemic attack, stroke, or any unexplained loss of consciousness within 1 year prior to screening or between screening and randomization.

8. Had presence of any psychiatric disorder according to the criteria of the Diagnostic and Statistical Manual of Mental Disorders, 5th Edition (DSM-V)1 or symptom if, in the judgment of the investigator, the psychiatric disorder or symptom was likely to confound interpretation of the study results, affect motor function assessment, or affect the participant’s ability to complete the study.

9. Had a diagnosis of intellectual disability (intellectual developmental disorder), mental retardation, or significant inherited cognitive impairment.

10. Suicidality, represented by answering “yes” to Question 4 or Question 5 on the Columbia Suicide Severity Rating Scale (C-SSRS), indicating active suicidal ideation with any intent to act, during the participant’s lifetime, as assessed at screening, or between screening and randomization.

11. Suicidal behaviour such that a determination of “yes” was made on the Suicidal Behaviour section of the C-SSRS for “Actual Attempt,” “Interrupted Attempt,” “Aborted Attempt,” or “Preparatory Acts or Behaviour,” during the participant’s lifetime, as assessed at screening, or between screening and randomization.

12. Had history of alcohol or drug abuse or dependence (except nicotine dependence), as defined by the Diagnostic and Statistical Manual of Mental Illnesses 5, within 2 years prior to screening or between screening and randomization.

13. Within 1 year prior to screening, had any of the following: myocardial infarction; hospitalization for congestive heart failure; hospitalization for, or symptoms of unstable angina; unexplained syncope.

14. Had moderate or severe congestive heart failure or known ejection fraction <40%.

15. Known significant structural heart disease (e.g., significant valvular disease, hypertrophic cardiomyopathy) that was considered likely to lead to a deterioration of cardiac function over the course of the study.

16. Had history of cancer within 5 years prior to screening or between screening and randomization, with the exception of non-metastatic basal and/or squamous cell carcinoma of the skin.

17. Had history of allergy/hypersensitivity to immunizations or immunoglobulins.

18. Any condition that, in the opinion of the investigator or medical monitor, made the participant unsuitable for the study.

*Prior/Concomitant Therapy*

19. Required treatment with another monoclonal antibody.

20. Used any investigational medicine, device, or biologic within 3 months or 5 half-lives of that intervention (whichever was longer) prior to screening.

21. Had undergone previous allogeneic bone marrow or stem cell transplant.

22. Used typical or atypical antipsychotic medication, or other medication with dopamine antagonist properties (e.g., metoclopramide, domperidone), within 6 months prior to randomization.

23. Used immunosuppressive medication within 6 months prior to randomization. (Note: Inhaled and topical corticosteroids were permitted. Low-dose systemic corticosteroids [<10 mg per day prednisone or equivalent], for autoimmune disease that was considered quiescent, in remission, or otherwise well controlled were permitted). Other immunosuppressive drugs and biologics were contraindicated.

24. Received non-leukocyte-depleted whole blood transfusion within 6 months prior to screening.

25. Received any commercially available vaccine within 30 days prior to randomization. (Note: for COVID-19 vaccines authorized by the FDA for emergency use, this timeframe applied from last vaccination or booster dose, whichever was required to consider vaccination complete in line with applicable guidance).

*Prior/Concurrent Clinical Study Experience*

26. Participated in another study investigating: (a) Active or passive immunization against α-synuclein for Parkinson’s disease, at any time prior to screening, or (b) Immunoglobulin G therapy within 6 months before screening.

*Diagnostic Assessments*

27. Any clinically significant abnormality as determined by investigator at screening or between screening and randomization in physical examination, vital signs, ECG, or clinical laboratory test results that may have compromised the participant’s safety or ability to complete the study or compromise the interpretation of the study results.

28. Presence of any of the following MRI contraindications: pacemaker; cardiac defibrillator; spinal cord or vagus nerve stimulator; aneurysm clip; artificial heart valve; recent (within 1 year) coronary or carotid stent; ear implant; CSF shunt; other implanted medical device (e.g., insulin pump); metal fragments or foreign objects in the eyes, skin, or body; claustrophobia which may have contraindicated a brain MRI scan.

29. Brain MRI findings (or historical radiologic reports, if available) that showed evidence of clinically significant structural brain disease which, in the opinion of the investigator, contraindicated the performance of lumbar puncture.

30. Any spinal abnormality or other aspects (e.g., tattoos) or other clinical findings (papilledema seen with ophthalmoscopy) that may have complicated or contraindicated lumbar puncture, as judged by the investigator.

31. Ophthalmic abnormalities. The following were considered exclusionary:

a) Congenital or acquired ophthalmic conditions (primary or secondary) that were considered poorly controlled within the last 12 months prior to screening, with or without treatment, or otherwise expected to lead to significant deterioration in visual acuity in the next 6 months after randomization.

b) Specific ophthalmic conditions:

i. Current or past history of inflammation affecting the uveal tract or sclera

ii. Diabetic retinopathy

iii. Neovascular or exudative (wet) form of age-related macular degeneration

iv. Active central serous retinopathy (central serous chorioretinopathy)

v. Participants with active autoimmune disease

vi. Participants who had taken immunosuppressive drugs (other than low doses of systemic steroids [<10 mg per day of prednisone equivalent], for autoimmune disease that was considered inactive, in remission, or otherwise well-controlled). Other immunosuppressive drugs were contraindicated.

vii. Mature cataracts (Grade ≥4 per the Lens Opacities Classification System III) or lower grade cataracts that were otherwise considered by the examining ophthalmologist to prevent adequate examination of the uveal tract or posterior ocular segment. (Note: Previous cataract surgery or an age-related cataract with age-appropriate lens opacification and no other important identified secondary causes [e.g., diabetes, corticosteroids, vitamin deficiencies, trauma, radiation, or systemic fluid and electrolyte disturbances] was not necessarily exclusionary).

32. Aspartate aminotransferase or alanine aminotransferase concentrations >1.5 × the upper limit of normal (ULN) at screening, or between screening and baseline.

33. Estimated creatinine clearance <50mL/min or 1.5 × ULN at screening.

34. Clinically significant vital signs abnormalities at screening or on Day 1, defined as (a) systolic blood pressure ≥160 mmHg, (b) diastolic blood pressure ≥90 mmHg (blood pressure assessed at rest; may be repeated up to 3 times), or (c) pulse rate <45 or >100 beats per minute (at rest).

35. Clinically significant abnormality in ECG rhythm, conduction, or morphology at screening or between screening and randomization, including but not limited to:

a) Clinically significant PR (PQ) interval prolongation (PR >220 msec)

b) Intermittent second- or third-degree atrioventricular (AV) block (AV block II Mobitz Type I, Wenckebach, while asleep or in deep rest was not exclusionary)

c) Bundle branch block or intraventricular conduction delay with QRS interval duration ≥120 msec

d) Electrocardiogram interval measured from the onset of the QRS complex to the end of the T wave interval corrected for heart rate using Fridericia’s formula interval measurement >470 msec, or a shortened QTcF <340 msec, at screening or between screening and randomization, or a family history of long or short QT syndrome.

36. Positive serologic findings for human immunodeficiency virus (HIV) antibodies, hepatitis B surface antigen, or hepatitis C virus antibodies, with relevant confirmatory testing conducted, where applicable, in accordance with Centers for Disease Control and Prevention guidance for HIV and viral hepatitis.

37. Current blood clotting or bleeding disorder, including clinically significant abnormal findings in laboratory tests of coagulation.

*Other Exclusions*

38. Poor venous access, such that IV drug delivery or pharmacokinetic/safety blood sampling was difficult.

39. Donated blood or plasma within 2 months prior to screening and until 2 months after the final follow-up visit.

40. A positive serum pregnancy test result at screening or prior to randomization.

41. Urine drug screen positive for a drug of abuse (except for permitted, prescribed opiates and/or benzodiazepines). A urine drug screen positive for cannabinoids was exclusionary unless there was a documented legitimate medical reason for the participant’s cannabinoid use (e.g., chronic pain) or the investigator and medical monitor agreed that the participant could abstain from use for the duration of the study.

42. Currently employed by the sponsor (AstraZeneca) or by a contract research organization or clinical study site participating in this study, or a first-degree relative of an AstraZeneca employee or of an employee at a participating contract research organization or clinical study site.

**Randomization**

Participants were assigned unique randomization codes in sequential order as they became eligible according to a schedule provided by clinical Contract Research Organisation (Covance Inc) for the SAD study and by an Interactive Voice/Web Response System (EndPoint Clinical) in the MAD study. In both studies, a sentinel dosing approach was used for the first two participants in each cohort randomized 1:1 to MEDI1341 or placebo. In the SAD study the remaining six participants were randomized to MEDI1341 or placebo in an 5:1 ratio, and in the MAD study the remaining 10 participants were randomized to MEDI1341 or placebo in an 8:2 ratio.

**Physical and neurological examinations**

The physical examination included an assessment of general appearance and a review of the following systems: cardiovascular, chest/lungs/respiratory, gastrointestinal, thyroid/neck, lymphatics, dermatological/skin, musculoskeletal/extremities, neurological, and head/ears/eyes/nose/throat (including mouth).

The neurologic examination included an assessment of mental status, cranial nerves (including pupil equality and reactivity), motor system, sensory system, cerebellar function, gait, and reflexes.

**Ophthalmic assessments**

The following examinations were performed by a licensed specialist/technician who was appropriately trained to undertake these diagnostic procedures:

- Standardized ophthalmic patient-reported outcome (PRO) questionnaire (National Eye Institute - Visual Functioning Questionnaire-25 [NEI-VFQ-25])
- Medical and surgical history review relevant to ophthalmology
- Visual acuity at distance (with best corrected vision)
- Automated visual field assessments
- Tonometry for intraocular pressure.
- Pachymetry for corneal thickness
- External and slit-lamp examination for eyelids, lacrimal system, motility, conjunctiva/sclera, limbus, cornea, anterior chamber, iris
- Dilated eye fundoscopy for posterior chamber, vitreous, dilated and fundus examination for optic disc, macula, blood vessels
- Optical coherence tomography to provide for high-resolution examination of the anterior eye segment and the posterior eye segment, to include a retinal macular scan and disc analysis, with measurement of retinal nerve fibre layer thickness and choroidal thickness

Clinical ophthalmic examinations were performed by a suitably trained and qualified practitioner in accordance with accepted local standards of ophthalmic care. All relevant qualitative findings (including findings of “no abnormality detected”) and quantitative measurements were documented in the ophthalmic source documents. At the follow-up (post-dose) assessment, it was clearly documented whether there had been any clinically significant changes from baseline.

Ophthalmic assessments were conducted on 2 occasions in the SAD: at screening and at follow-up (no earlier than Day 57 and no later than the final follow-up visit). Intraocular pressure was also measured at Day 29 (Visit 7), in addition to the assessment of visual acuity and administration of the NEI-VFQ-25. In the MAD, ophthalmic assessments were conducted at screening, Day 15, Day 43 and Day 85. NEI-VFQ-25 was administered at screening and on Days -1, 3, 15, 29, 43, 57/58, 64, 85 and 148.

**Quantitation of MEDI1341 in Human Serum Using a Validated Electrochemiluminescent Detection Assay**

The concentration of MEDI1341 in human serum was measured using a sandwich immunoassay in which a capture antibody (biotinylated anti-MEDI1341) was immobilized in a 96-well plate, analyte was bound to the capture antibody, and a ruthenylated (Sulfo-TAG) conjugated secondary detection antibody (ruthenylated-Streptavidin) was used to generate an electrochemiluminescent response. The electrochemiluminescent response generated was directly proportional to the amount of analyte detected in the well. Back-calculated concentrations (BCC) of analyte were generated using a 5 parameter logistic (PL) fit model with 1/Y weighting.

**Quantitation of MEDI1341 in Human CSF Using a Validated Electrochemiluminescent Detection Assay**

The concentration of MEDI1341 in human CSF was measured using a sandwich immunoassay in which a capture antibody (BT-anti-MEDI1341) was immobilized in a 96- well plate, analyte was bound to the capture antibody, and a secondary detection antibody (Sulfo-TAG-anti-MEDI1341) was used to generate an absorbance response. The electrochemiluminescent response generated was directly proportional to the amount of analyte detected in the well. BCC of analyte were generated using a 4PL fit model with 1/Y^2^ weighting.

**Determination of free α-synuclein in human CSF**

The concentration of free α-synuclein in human CSF was determined using a U-Plex human α-synuclein kit (Cat. K151WKK) from MSD utilizing 100 µL of sample for duplicate analysis (minimum required dilution of 1 in 2, 25 µL added to each well). The α-synuclein bound to MEDI1341 was removed by immunoprecipitation prior to analysis. The analytical method was validated at York Bioanalytical Solutions prior to sample analysis. The concentration of human IgG (huIgG) in human CSF, before and after immunoprecipitation, was determined using a huIgG electrochemiluminescent assay (Cat. F160) from Cygnus Technologies to confirm successful removal of MEDI1341 antibodies. The concentration of haemoglobin in CSF was determined using a Haemoglobin Assay Kit (Cat. MAK115) from Sigma-Aldrich.

**Determination of total α-synuclein in human plasma**

The concentration of total α-synuclein in human plasma was determined using a U-Plex human α-synuclein kit (Cat. K151WKK) from MSD utilizing 20 µL of sample for duplicate analysis (minimum required dilution of 1 in 20, 25 µL added to each well). The analytical method was validated at York Bioanalytical Solutions prior to sample analysis.

**Determination of antidrug antibodies (ADAs) in human serum**

Presence of ADA in human serum was determined using the Meso Scale Discovery (MSD) electrochemiluminescence platform. Biotin-conjugated MEDI1341 was used to capture anti-MEDI1341 antibodies which were subsequently detected with Sulfo TAG-conjugated MEDI1341, using an MSD S600 plate reader. The presence of ADA was determined by comparing the signal in the sample to a statistically derived threshold.

Negative results were those that were either negative in the screening assay and the confirmatory assay or were positive in the screening assay but negative in the confirmatory assay. Borderline positive results were positive in both the screening and confirmatory assays, but with no titres obtained (titres were reported as <50). Positive results were positive in both the screening and confirmatory assays, with a titre ≥50 obtained.

**Assessment of dose proportionality and linearity**

In the SAD study, dose proportionality and linearity were evaluated using a power model. If linearity was not seen, log-transformed, dose-normalized pharmacokinetic parameters were input into an analysis of variance (ANOVA) model which included treatment dose as a fixed effect. P-values for overall and pairwise treatment comparisons were calculated. As only two dose levels were evaluated in the MAD study, dose proportionality and linearity were only assessed by log-transformed, dose-normalized pharmacokinetic parameters inputted into an ANOVA model which included treatment dose as a fixed effect.

**Supplementary Figure 1.** Mean (SD) Serum concentrations of MEDI1341 following (A) single IV dose of MEDI1341 in healthy participants and (B) multiple IV doses after third infusion (Day 57) in participants with Parkinson’s disease


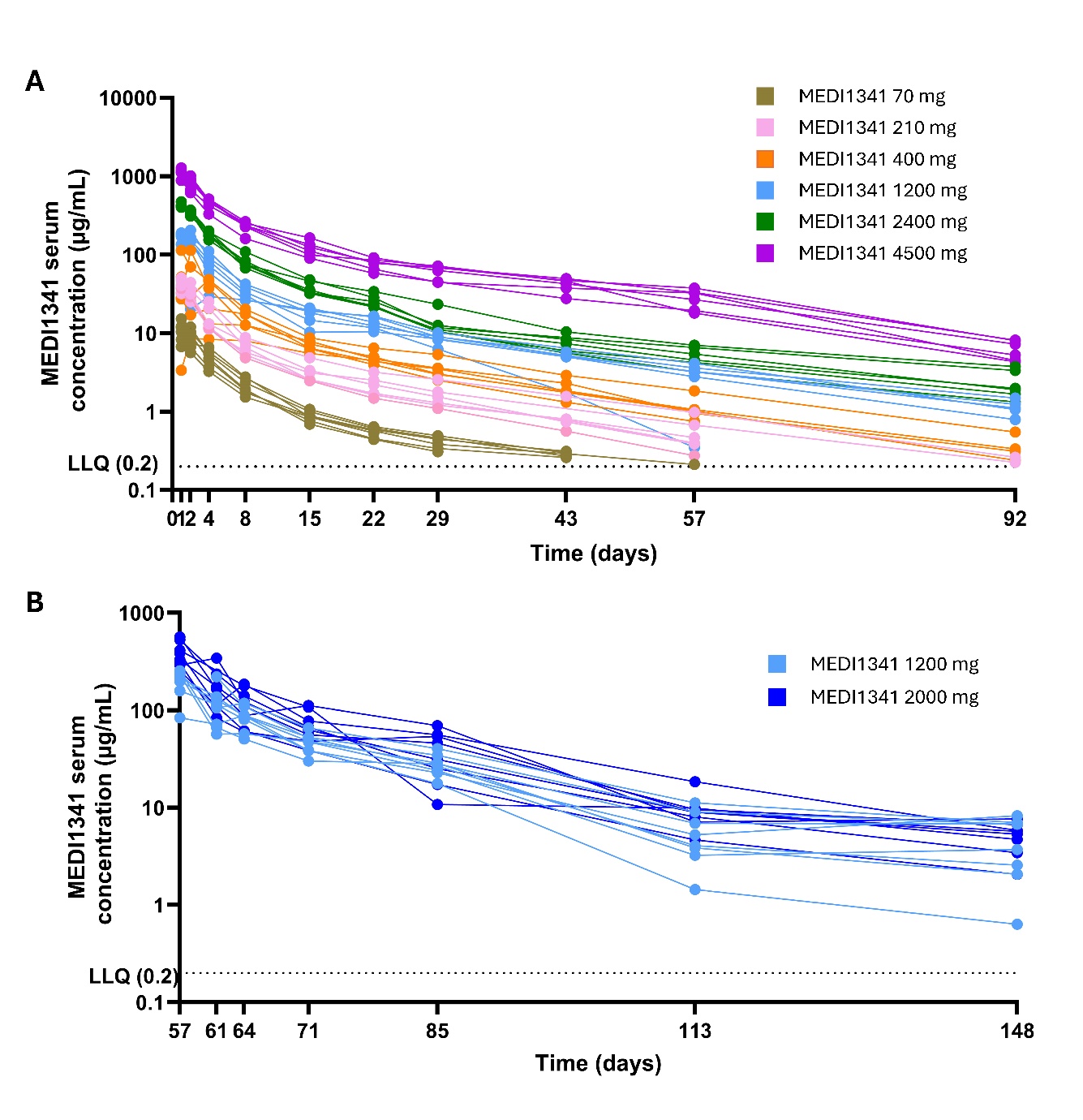


**IV**, intravenous; **LLQ**, lower limit of quantitation; **SD**, standard deviation.

N=6 for each dose cohort and N=13 for placebo in the SAD. N=9 for each dose cohort and N=7 for placebo in the MAD.**Supplementary Figure 2.** Change from baseline in (A) total α-synuclein concentration in plasma and (B) CSF free α-synuclein concentration following single MEDI1341 IV dose in healthy participants


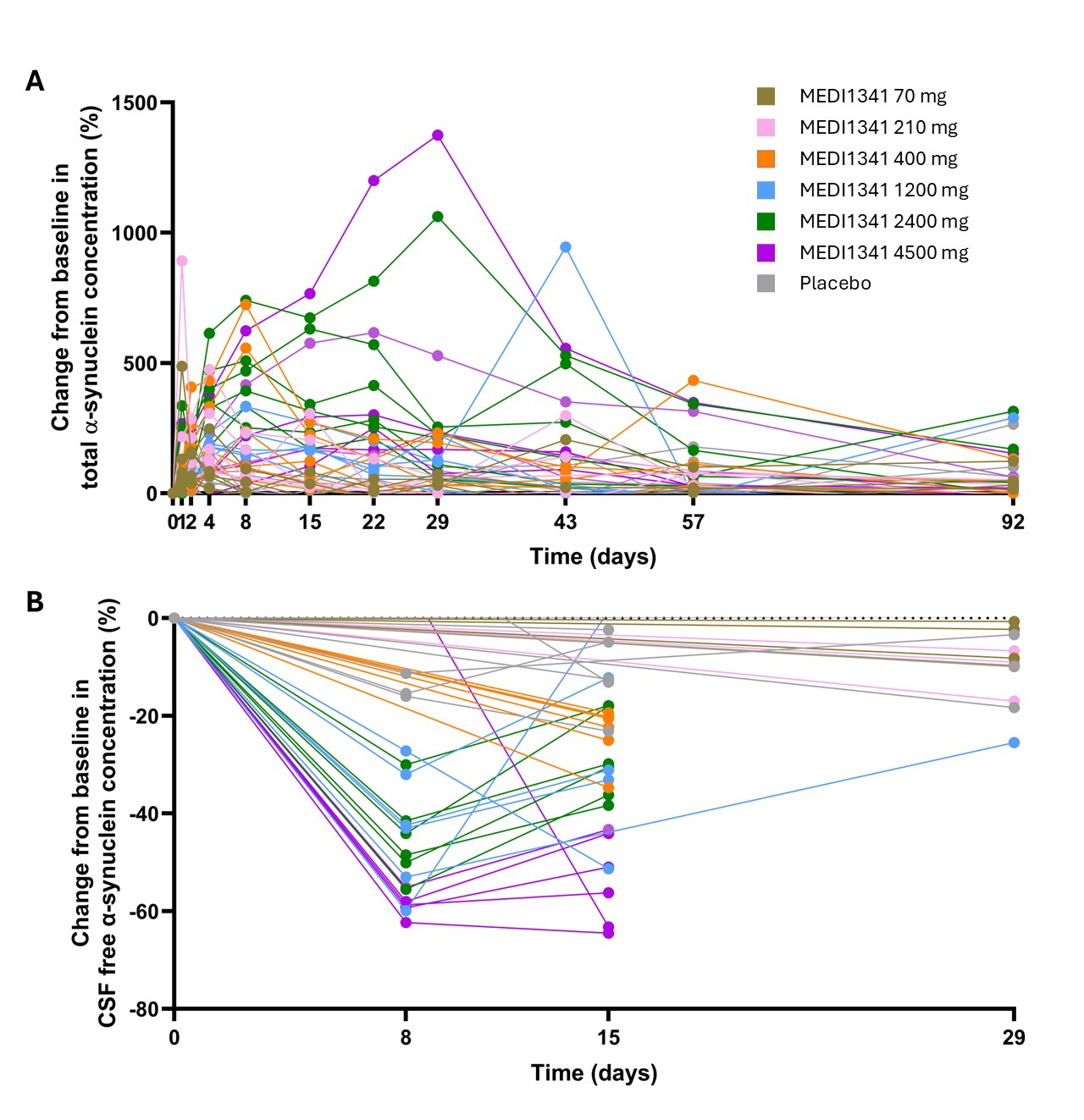

**CSF**, cerebrospinal fluid; **IV**, intravenous.

N=6 for each dose cohort and N=13 for placebo in the SAD. N=9 for each dose cohort and N=7 for placebo in the MAD.

**Supplementary Figure 3.** Change from baseline following multiple MEDI1341 IV doses in participants with Parkinson’s disease in (A) total α-synuclein concentration in plasma (following the last administered dose [Day 57]) and (B) CSF free α-synuclein concentration


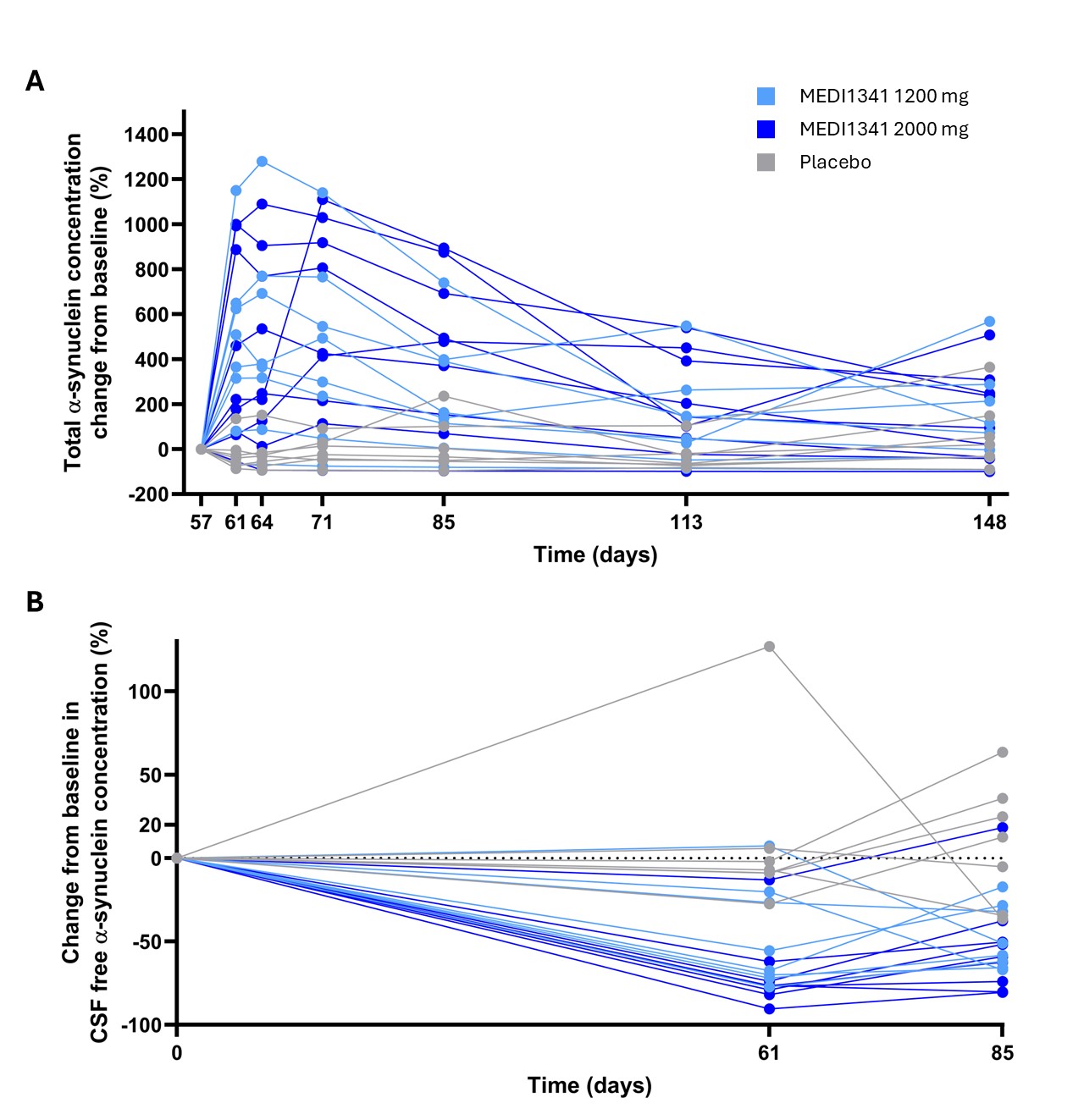


**CSF**, cerebrospinal fluid; **IV**, intravenous.

N=6 for each dose cohort and N=13 for placebo in the SAD. N=9 for each dose cohort and N=7 for placebo in the MAD.

**Supplementary Table 1.** Blood and CSF sampling for healthy participants

| **Visit description** | **Screening** | **Treatment** | | | | **Follow-up** | | | | | | |
| --- | --- | --- | --- | --- | --- | --- | --- | --- | --- | --- | --- | --- |
|  |  | **Inpatient^a^** | | |  |  |  |  |  |  |  |  |
| **Visit number** | **1** | **2** | | | **3** | **4** | **5** | **6** | **7** | **8** | **9** | **10^b^** |
| **Study day** | **-49 to -2** | **-1** | **1** | **2** | **4 (± 1 day)** | **8 (± 1 day)** | **15 (± 2 days)** | **22 (± 2 days)** | **29 (± 2 days)** | **43 (± 3 days)** | **57 (± 3 days)** | **92 (± 3 days)** |
| **Blood sampling for** |  |  |  |  |  |  |  |  |  |  |  |  |
| PK of MEDI1341l |  |  | X | X | X | X | X | X | X | X | X | X |
| ADA |  |  | X |  |  |  | X |  | X |  | X | X |
| α-synuclein (total) |  |  | X | X | X | X | X | X | X | X | X | X |
| **CSF sampling for** |  |  |  |  |  |  |  |  |  |  |  |  |
| PK of MEDI1341 |  | X |  |  |  | X | X |  |  |  |  |  |
| α-synuclein (free) |  | X |  |  |  | X | X |  | X |  |  |  |
| Bioanalytical methodology |  | X |  |  |  |  |  |  |  |  |  |  |
| **Vital signs** | X | X | X | X | X | X | X | X | X | X | X | X |
| **Physical examination** | X | X |  |  |  | X | X |  | X |  |  | X |
| **Neurological examination** | X | X |  |  |  | X | X |  | X |  |  | X |
| **Paper 12-lead ECG** | X | X | X | X |  | X |  |  | X |  | X | X |
| **Digital 12-lead ECG** |  |  | X | X |  | X |  |  | X |  | X | X |
| **ECG telemetry** |  | X | X | X |  |  |  |  |  |  |  |  |

**ADA**, antidrug antibody; **CRU**, clinical research unit; **CSF**, cerebrospinal fluid; **ECG**, electrocardiogram; **PK**, pharmacokinetic

^a^Participants entered the CRU on the day prior to infusion administration and remained in the CRU until at least 24 hours after end of infusion; ^b^Participants who withdrew/were withdrawn from the study before completion should have returned to the CRU as soon as possible after withdrawal and underwent the assessments specified for this final visit. If the participant withdrew before the Day 29 visit, a CSF sample should have been obtained if possible

**Supplementary Table 2.** Blood and CSF sampling for participants with Parkinson’s disease

| **Visit description** | **Screening** | **Treatment** | | | | | | | | | **Follow-up** | | | | | |
| --- | --- | --- | --- | --- | --- | --- | --- | --- | --- | --- | --- | --- | --- | --- | --- | --- |
|  |  | **(inpatient)^a^** | | | **Outpatient** | | | | | |  |  |  |  |  |  |
| **Visit number** | **1** | **2** | | | **3** | **4** | **5** | **6** | **7** | **8** | **9** | **10** | **11** | **12** | **13** | **14^b^** |
| **Study day** | **-49 to -2** | **-1** | **1** | **2** | **4 (± 1 day)** | **8 (± 2 day)** | **15 (± 2 days)** | **29 (± 2 days)** | **43 (± 2 days)** | **57/58 (± 2 days)** | **61 (± 2 days)** | **64 (± 2 days)** | 71 (± 3 days) | 85 (± 3 days) | 113 (± 4 days) | 148 (± 7 days) |
| **Blood sampling for** |  |  |  |  |  |  |  |  |  |  |  |  |  |  |  |  |
| PK of MEDI1341l |  |  | X | X | X | X | X | X |  | X | X | X | X | X | X | X |
| ADA |  |  | X |  |  |  | X | X |  | X |  |  | X | X | X | X |
| α-synuclein (total) |  |  | X | X | X | X | X | X |  | X | X | X | X | X | X | X |
| **CSF sampling for** |  |  |  |  |  |  |  |  |  |  |  |  |  |  |  |  |
| PK of MEDI1341 | X |  |  |  |  |  |  |  |  |  | X |  |  | X |  |  |
| α-synuclein (free) | X |  |  |  |  |  |  |  |  |  | X |  |  | X |  |  |
| Bioanalytical methodology | X |  |  |  |  |  |  |  |  |  | X |  |  | X |  |  |
| **Vital signs** | X | X | X | X | X | X | X | X | X | X | X | X | X | X | X | X |
| **Physical examination** | X | X |  |  |  | X |  | X | X | X |  |  | X | X | X | X |
| **Neurological examination** | X | X |  |  |  | X |  | X | X | X |  |  | X | X | X | X |
| **Paper 12-lead ECG** | X | X | X | X |  | X | X | X | X | X |  |  | X | X | X | X |
| **Digital 12-lead ECG** |  |  | X | X |  | X |  | X |  | X |  |  |  | X | X | X |
| **ECG telemetry** |  | X | X | X |  |  |  | X |  | X |  |  |  |  |  |  |

**ADA**, antidrug antibody; **CRU**, clinical research unit; **CSF**, cerebrospinal fluid; **ECG**, electrocardiogram; **PK**, pharmacokinetic

^a^For Visit 2 (first infusion), participants entered the CRU on the day prior to infusion administration and remained in the CRU until at least 24 hours after the end of the infusion. For Visit 6 (second infusion), and Visit 8 (third infusion), participants entered the CRU on the day of the infusion and remained in the CRU for at least 8 hours following the infusion for post infusion assessments. Participants may have remained in the CRU for up to 24 hours following the infusion, based on the investigator’s judgment. For Visit 8 (third infusion), participants who left the CRU after the 8-hour assessments returned to the CRU for the 24-hour post infusion assessments (PK and vital signs); ^b^Participants who withdrew/were withdrawn from the study before completion returned to the CRU as soon as possible after withdrawal and the assessments specified for this final visit were performed. If the participant withdrew after completing the third infusion and before the Day 85 visit, a CSF sample was obtained as possible.

**Supplementary Table 3.** Frequency of TEAEs by system organ class and preferred term in healthy participants

| **Demographic** | **Placebo (N=13)** | **70 mg (N=6)** | **210 mg (N=6)** | **400 mg (N=6)** | **1200 mg (N=6)** | **2400 mg (N=6)** | **4500 mg (N=6)** | **Overall (N=49)** |
| --- | --- | --- | --- | --- | --- | --- | --- | --- |
| **Overall** | 6 (46.2) | 1 (16.7) | 1 (16.7) | 4 (66.7) | 6 (100.0) | 3 (50.0) | 2 (33.3) | 23 (46.9) |
| **Injury, poisoning, and procedural complications** | 6 (46.2) | 1 (16.7) | 0 (0.0) | 1 (16.7) | 3 (50.0) | 0 (0.0) | 1 (16.7) | 12 (24.5) |
| Procedural site reaction | 3 (23.1) | 0 (0.0) | 0 (0.0) | 0 (0.0) | 1 (16.7) | 0 (0.0) | 1 (16.7) | 5 (10.2) |
| Post lumbar puncture syndrome | 1 (7.7) | 0 (0.0) | 0 (0.0) | 1 (16.7) | 2 (33.3) | 0 (0.0) | 0 (0.0) | 4 (8.2) |
| Procedure pain | 2 (15.4) | 1 (16.7) | 0 (0.0) | 0 (0.0) | 0 (0.0) | 0 (0.0) | 0 (0.0) | 3 (6.1) |
| Post procedural contusion | 1 (7.7) | 0 (0.0) | 0 (0.0) | 0 (0.0) | 1 (16.7) | 0 (0.0) | 0 (0.0) | 2 (4.1) |
| Procedural headache | 2 (15.4) | 0 (0.0) | 0 (0.0) | 0 (0.0) | 0 (0.0) | 0 (0.0) | 0 (0.0) | 2 (4.1) |
| Post procedural swelling | 1 (7.7) | 0 (0.0) | 0 (0.0) | 0 (0.0) | 0 (0.0) | 0 (0.0) | 0 (0.0) | 1 (2.0) |
| Thermal burn | 0 (0.0) | 0 (0.0) | 0 (0.0) | 0 (0.0) | 0 (0.0) | 0 (0.0) | 1 (16.7) | 1 (2.0) |
| **Nervous system disorders** | 0 (0.0) | 0 (0.0) | 1 (16.7) | 2 (33.3) | 4 (66.7) | 1 (16.7) | 1 (16.7) | 9 (18.4) |
| Headache | 0 (0.0) | 0 (0.0) | 1 (16.7) | 2 (33.3) | 4 (66.7) | 0 (0.0) | 1 (16.7) | 8 (16.3) |
| Dysgeusia | 0 (0.0) | 0 (0.0) | 0 (0.0) | 0 (0.0) | 0 (0.0) | 1 (16.7) | 0 (0.0) | 1 (2.0) |
| Paraesthesia | 0 (0.0) | 0 (0.0) | 0 (0.0) | 1 (16.7) | 0 (0.0) | 0 (0.0) | 0 (0.0) | 1 (2.0) |
| Somnolence | 0 (0.0) | 0 (0.0) | 0 (0.0) | 0 (0.0) | 1 (16.7) | 0 (0.0) | 0 (0.0) | 1 (2.0) |
| **Gastrointestinal disorders** | 1 (7.7) | 0 (0.0) | 1 (16.7) | 2 (33.3) | 0 (0.0) | 0 (0.0) | 1 (16.7) | 5 (10.2) |
| Nausea | 1 (7.7) | 0 (0.0) | 1 (16.7) | 2 (33.3) | 0 (0.0) | 0 (0.0) | 1 (16.7) | 5 (10.2) |
| Vomiting | 1 (7.7) | 0 (0.0) | 1 (16.7) | 1 (16.7) | 0 (0.0) | 0 (0.0) | 0 (0.0) | 3 (6.1) |
| Dysphagia | 0 (0.0) | 0 (0.0) | 0 (0.0) | 1 (16.7) | 0 (0.0) | 0 (0.0) | 0 (0.0) | 1 (2.0) |
| **Musculoskeletal and connective tissue disorders** | 0 (0.0) | 0 (0.0) | 0 (0.0) | 2 (33.3) | 1 (16.7) | 2 (33.3) | 0 (0.0) | 5 (10.2) |
| Back pain | 0 (0.0) | 0 (0.0) | 0 (0.0) | 1 (16.7) | 0 (0.0) | 1 (16.7) | 0 (0.0) | 2 (4.1) |
| Arthralgia | 0 (0.0) | 0 (0.0) | 0 (0.0) | 0 (0.0) | 1 (16.7) | 0 (0.0) | 0 (0.0) | 1 (2.0) |
| Groin pain | 0 (0.0) | 0 (0.0) | 0 (0.0) | 0 (0.0) | 1 (16.7) | 0 (0.0) | 0 (0.0) | 1 (2.0) |
| Musculoskeletal stiffness | 0 (0.0) | 0 (0.0) | 0 (0.0) | 1 (16.7) | 0 (0.0) | 0 (0.0) | 0 (0.0) | 1 (2.0) |
| Myofascial pain syndrome | 0 (0.0) | 0 (0.0) | 0 (0.0) | 0 (0.0) | 0 (0.0) | 1 (16.7) | 0 (0.0) | 1 (2.0) |
| **Investigations** | 1 (7.7) | 0 (0.0) | 0 (0.0) | 0 (0.0) | 1 (16.7) | 0 (0.0) | 1 (16.7) | 3 (6.1) |
| Aspartate aminotransferase increased | 1 (7.7) | 0 (0.0) | 0 (0.0) | 0 (0.0) | 0 (0.0) | 0 (0.0) | 1 (16.7) | 2 (4.1) |
| Blood creatine phosphokinase increased | 1 (7.7) | 0 (0.0) | 0 (0.0) | 0 (0.0) | 0 (0.0) | 0 (0.0) | 1 (16.7) | 2 (4.1) |
| Weight increased | 0 (0.0) | 0 (0.0) | 0 (0.0) | 0 (0.0) | 1 (16.7) | 0 (0.0) | 0 (0.0) | 1 (2.0) |
| **Eye disorders** | 1 (7.7) | 0 (0.0) | 0 (0.0) | 1 (16.7) | 1 (16.7) | 0 (0.0) | 0 (0.0) | 3 (6.1) |
| Asthenopia | 0 (0.0) | 0 (0.0) | 0 (0.0) | 1 (16.7) | 0 (0.0) | 0 (0.0) | 0 (0.0) | 1 (2.0) |
| Dry eye | 0 (0.0) | 0 (0.0) | 0 (0.0) | 0 (0.0) | 1 (16.7) | 0 (0.0) | 0 (0.0) | 1 (2.0) |
| Vision blurred | 1 (7.7) | 0 (0.0) | 0 (0.0) | 0 (0.0) | 0 (0.0) | 0 (0.0) | 0 (0.0) | 1 (2.0) |
| **General disorders and administration site conditions** | 0 (0.0) | 0 (0.0) | 0 (0.0) | 1 (16.7) | 0 (0.0) | 1 (16.7) | 1 (16.7) | 3 (6.1) |
| Chest discomfort | 0 (0.0) | 0 (0.0) | 0 (0.0) | 1 (16.7) | 0 (0.0) | 0 (0.0) | 0 (0.0) | 1 (2.0) |
| Feeling hot | 0 (0.0) | 0 (0.0) | 0 (0.0) | 0 (0.0) | 0 (0.0) | 0 (0.0) | 1 (16.7) | 1 (2.0) |
| Pyrexia | 0 (0.0) | 0 (0.0) | 0 (0.0) | 0 (0.0) | 0 (0.0) | 1 (16.7) | 0 (0.0) | 1 (2.0) |
| **Infections and infestations** | 1 (7.7) | 0 (0.0) | 0 (0.0) | 0 (0.0) | 2 (33.3) | 0 (0.0) | 0 (0.0) | 3 (6.1) |
| Influenza | 0 (0.0) | 0 (0.0) | 0 (0.0) | 0 (0.0) | 1 (16.7) | 0 (0.0) | 0 (0.0) | 1 (2.0) |
| Sinusitis | 1 (7.7) | 0 (0.0) | 0 (0.0) | 0 (0.0) | 0 (0.0) | 0 (0.0) | 0 (0.0) | 1 (2.0) |
| Viral upper respiratory tract infection | 0 (0.0) | 0 (0.0) | 0 (0.0) | 0 (0.0) | 1 (16.7) | 0 (0.0) | 0 (0.0) | 1 (2.0) |
| **Skin and subcutaneous tissue disorders** | 1 (7.7) | 0 (0.0) | 0 (0.0) | 1 (16.7) | 0 (0.0) | 0 (0.0) | 0 (0.0) | 2 (4.1) |
| Dermatitis contact | 1 (7.7) | 0 (0.0) | 0 (0.0) | 0 (0.0) | 0 (0.0) | 0 (0.0) | 0 (0.0) | 1 (2.0) |
| Pruritus | 0 (0.0) | 0 (0.0) | 0 (0.0) | 1 (16.7) | 0 (0.0) | 0 (0.0) | 0 (0.0) | 1 (2.0) |
| **Blood and lymphatic system disorders** | 0 (0.0) | 0 (0.0) | 0 (0.0) | 1 (16.7) | 0 (0.0) | 0 (0.0) | 0 (0.0) | 1 (2.0) |
| Lymphadenopathy | 0 (0.0) | 0 (0.0) | 0 (0.0) | 1 (16.7) | 0 (0.0) | 0 (0.0) | 0 (0.0) | 1 (2.0) |
| **Cardiac disorders** | 0 (0.0) | 0 (0.0) | 0 (0.0) | 1 (16.7) | 0 (0.0) | 0 (0.0) | 0 (0.0) | 1 (2.0) |
| Tachyarrhythmia | 0 (0.0) | 0 (0.0) | 0 (0.0) | 1 (16.7) | 0 (0.0) | 0 (0.0) | 0 (0.0) | 1 (2.0) |
| **Ear and labyrinth disorders** | 0 (0.0) | 0 (0.0) | 0 (0.0) | 0 (0.0) | 1 (16.7) | 0 (0.0) | 0 (0.0) | 1 (2.0) |
| Cerumen impaction | 0 (0.0) | 0 (0.0) | 0 (0.0) | 0 (0.0) | 1 (16.7) | 0 (0.0) | 0 (0.0) | 1 (2.0) |
| **Metabolism and nutrition disorders** | 0 (0.0) | 0 (0.0) | 0 (0.0) | 0 (0.0) | 1 (16.7) | 0 (0.0) | 0 (0.0) | 1 (2.0) |
| Increased appetite | 0 (0.0) | 0 (0.0) | 0 (0.0) | 0 (0.0) | 1 (16.7) | 0 (0.0) | 0 (0.0) | 1 (2.0) |
| **Respiratory, thoracic and mediastinal disorders** | 0 (0.0) | 0 (0.0) | 0 (0.0) | 0 (0.0) | 1 (16.7) | 0 (0.0) | 0 (0.0) | 1 (2.0) |
| Rhinitis allergic | 0 (0.0) | 0 (0.0) | 0 (0.0) | 0 (0.0) | 1 (16.7) | 0 (0.0) | 0 (0.0) | 1 (2.0) |
| **Vascular disorders** | 0 (0.0) | 0 (0.0) | 0 (0.0) | 0 (0.0) | 1 (16.7) | 0 (0.0) | 0 (0.0) | 1 (2.0) |
| Peripheral coldness | 0 (0.0) | 0 (0.0) | 0 (0.0) | 0 (0.0) | 1 (16.7) | 0 (0.0) | 0 (0.0) | 1 (2.0) |

Data are reported as n (%) where n represents the number of participants with an adverse event.

**TEAE**, treatment-emergent adverse event

**Supplementary Table 4.** Frequency of TEAEs by system organ class and preferred term in participants with Parkinson’s disease

| **System Organ Class**  Preferred Term | **Placebo (N=7)** | **1200 mg (N=9)** | **2000 mg (N=9)** | **Overall (N=25)** |
| --- | --- | --- | --- | --- |
| **Overall** | 1 (14.3) | 4 (44.4) | 5 (55.6) | 10 (40.0) |
| **Gastrointestinal disorders** | 1 (14.3) | 1 (11.1) | 2 (22.2) | 4 (16.0) |
| Nausea | 0 (0.0) | 1 (11.1) | 1 (11.1) | 2 (8.0) |
| Diarrhoea | 0 (0.0) | 0 (0.0) | 1 (11.1) | 1 (4.0) |
| Large intestine polyp | 0 (0.0) | 0 (0.0) | 1 (11.1) | 1 (4.0) |
| Toothache | 1 (14.3) | 0 (0.0) | 0 (0.0) | 1 (4.0) |
| **General disorders and administration site conditions** | 1 (14.3) | 2 (22.2) | 0 (0.0) | 3 (12.0) |
| Fatigue | 0 (0.0) | 1 (11.1) | 0 (0.0) | 1 (4.0) |
| Injection site discomfort | 0 (0.0) | 1 (11.1) | 0 (0.0) | 1 (4.0) |
| Tissue infiltration | 1 (14.3) | 0 (0.0) | 0 (0.0) | 1 (4.0) |
| **Infections and infestations** | 0 (0.0) | 2 (22.2) | 0 (0.0) | 2 (8.0) |
| Rhinitis | 0 (0.0) | 1 (11.1) | 0 (0.0) | 1 (4.0) |
| Upper respiratory tract infection | 0 (0.0) | 1 (11.1) | 0 (0.0) | 1 (4.0) |
| **Injury, poisoning and procedural complications** | 1 (14.3) | 1 (11.1) | 0 (0.0) | 2 (8.0) |
| Fall | 1 (14.3) | 1 (11.1) | 0 (0.0) | 2 (8.0) |
| Corneal abrasion | 0 (0.0) | 1 (11.1) | 0 (0.0) | 1 (4.0) |
| Skin abrasion | 0 (0.0) | 1 (11.1) | 0 (0.0) | 1 (4.0) |
| **Nervous system disorders** | 0 (0.0) | 1 (11.1) | 1 (11.1) | 2 (8.0) |
| Dizziness | 0 (0.0) | 0 (0.0) | 1 (11.1) | 1 (4.0) |
| Headache | 0 (0.0) | 1 (11.1) | 0 (0.0) | 1 (4.0) |
| Hemianopia heteronymous | 0 (0.0) | 1 (11.1) | 0 (0.0) | 1 (4.0) |
| **Respiratory, thoracic and mediastinal disorders** | 0 (0.0) | 2 (22.2) | 0 (0.0) | 2 (8.0) |
| Dyspnoea | 0 (0.0) | 1 (11.1) | 0 (0.0) | 1 (4.0) |
| Respiratory tract congestion | 0 (0.0) | 1 (11.1) | 0 (0.0) | 1 (4.0) |
| **Skin and subcutaneous tissue disorders** | 0 (0.0) | 1 (11.1) | 1 (11.1) | 2 (8.0) |
| Purpura senile | 0 (0.0) | 0 (0.0) | 1 (11.1) | 1 (4.0) |
| Rash | 0 (0.0) | 1 (11.1) | 0 (0.0) | 1 (4.0) |
| **Eye disorders** | 0 (0.0) | 0 (0.0) | 1 (11.1) | 1 (4.0) |
| Eye pruritus | 0 (0.0) | 0 (0.0) | 1 (11.1) | 1 (4.0) |
| **Immune system disorders** | 0 (0.0) | 1 (11.1) | 0 (0.0) | 1 (4.0) |
| Seasonal allergy | 0 (0.0) | 1 (11.1) | 0 (0.0) | 1 (4.0) |
| **Investigations** | 0 (0.0) | 0 (0.0) | 1 (11.1) | 1 (4.0) |
| Colonoscopy | 0 (0.0) | 0 (0.0) | 1 (11.1) | 1 (4.0) |
| **Musculoskeletal and connective tissue disorders** | 0 (0.0) | 1 (11.1) | 0 (0.0) | 1 (4.0) |
| Back pain | 0 (0.0) | 1 (11.1) | 0 (0.0) | 1 (4.0) |
| Pain in extremity | 0 (0.0) | 1 (11.1) | 0 (0.0) | 1 (4.0) |
| **Renal and urinary disorders** | 0 (0.0) | 0 (0.0) | 1 (11.1) | 1 (4.0) |
| Nephrolithiasis | 0 (0.0) | 0 (0.0) | 1 (11.1) | 1 (4.0) |

Data are reported as n (%) where n represents the number of participants with an adverse event

**TEAE**, treatment-emergent adverse event

**Supplementary Table 5.** Statistical assessment of dose proportionality of the serum pharmacokinetic parameters of MEDI1341 following single IV doses of MEDI1341 in healthy participants (Power Model)

| **Parameter** | **Slope** | **95% CI for the slope** | | **Pooled geometric CV% (between-subject)** | **Lack of fit 2-sided P-value** |
| --- | --- | --- | --- | --- | --- |
|  |  | **Lower** | **Upper** |  |  |
| **AUC_0–t_ (day*μg/mL)** | 1.11 | 1.06 | 1.17 | 20.9 | 0.0041~ |
| **AUC_0–∞_ (day*μg/mL)** | 1.10 | 1.04 | 1.16 | 20.7 | 0.0031~ |
| **C_max_ (μg/mL)** | 1.06 | 0.928 | 1.20 | 57.6 | 0.1040~ |

~ Assumption of linearity violated (lack of fit p-value ≤0.05)

Model: ln(parameter) = intercept + slope x ln(dose) + random error Lack of Fit Model: ln(parameter) = intercept + slope x ln(dose) + dose + random error

**AUC_0–t_**, area under the concentration-time curve from time zero to the last quantifiable concentration; **C_max_**, maximum observed concentration; **CI**, confidence interval; **CV%**, coefficient of variation

**Supplementary Table 6.** Statistical assessment of dose proportionality of the serum pharmacokinetic parameters of MEDI1341 following single IV doses of MEDI1341 in healthy participants (ANOVA model)

|  |  |  |  | **Pairwise 2-sided P-value** | | | | | |
| --- | --- | --- | --- | --- | --- | --- | --- | --- | --- |
| **Parameter** | **Dose** | **n** | **GLSM** | **70 mg** | **210 mg** | **400 mg** | **1200 mg** | **2400 mg** | **4500 mg** |
| Dose normalised AUC_0–t_ (day*μg/mL/mg) | Overall^a^ | 36 | <.0001* | - | - | - | - | - | - |
|  | 70 mg | 6 | 0.814 | - | 0.0707 | 0.1205 | 0.3956 | 0.0460* | <0.0001* |
|  | 210 mg | 6 | 1.02 | 0.0707 | - | 0.7843 | 0.3195 | 0.8367 | 0.0003* |
|  | 400 mg | 6 | 0.986 | 0.1205 | 0.7843 | - | 0.4674 | 0.6319 | 0.0001* |
|  | 1200 mg | 6 | 0.903 | 0.3956 | 0.3195 | 0.4674 | - | 0.2319 | <0.0001* |
|  | 2400 mg | 6 | 1.04 | 0.0460* | 0.8367 | 0.6319 | 0.2319 | - | 0.0006* |
|  | 4500 mg | 6 | 1.66 | <.0001* | 0.0003* | 0.0001* | <.0001* | 0.0006* | - |
| Dose normalised AUC_0–∞_ (day*μg/mL/mg) | Overall^a^ | 36 | 0.0001* | - | - | - | - | - | - |
|  | 70 mg | 6 | 0.916 | - | 0.2304 | 0.3812 | 0.9190 | 0.1752 | <0.0001* |
|  | 210 mg | 6 | 1.06 | 0.2304 | - | 0.7396 | 0.2709 | 0.8707 | 0.0004* |
|  | 400 mg | 6 | 1.02 | 0.3812 | 0.7396 | - | 0.4379 | 0.6210 | 0.0002* |
|  | 1200 mg | 6 | 0.927 | 0.9190 | 0.2709 | 0.4379 | - | 0.2083 | <0.0001* |
|  | 2400 mg | 6 | 1.08 | 0.1752 | 0.8707 | 0.6210 | 0.2083 | - | 0.0006* |
|  | 4500 mg | 6 | 1.70 | <.0001* | 0.0004* | 0.0002* | <.0001* | 0.0006* | - |

^a^The n and p-value from the overall dose comparison are presented

*p≤0.05

The GLSMs were obtained by taking the exponential of the LSMs on the natural log (ln) scale

**AUC_0–t_**, area under the concentration-time curve from time zero to the last quantifiable concentration; **GLSM**, geometric least squares mean; **IV**, intravenous; **LSM**, least square mean

**Supplementary Table 7.** CSF MEDI1341 concentrations and CSF/serum concentration ratios Following Single IV Doses of MEDI1341

| **Timepoint** | **70 mg**  **(N = 6)** | **210 mg**  **(N = 6)** | **400 mg**  **(N = 6)** | **1200 mg**  **(N = 6)** | **2400 mg**  **(N = 6)** | **4500 mg**  **(N = 6)** |
| --- | --- | --- | --- | --- | --- | --- |
| **Predose (ng/mL)** | - | - | - | - | - | - |
| **Day 8 (ng/mL)** | - | - | - | 76.6 (14.2) [6] | 142 (43.3) [6] | 407 (10.2) [6] |
| **Day 15 (ng/mL)** | - | - | 22.1 (16.2) [6] | 61.9 (20.7) [5] | 102 (55.9) [6] | 323 (17.2) [6] |
| **Day 29 (ng/mL)** | 2.4 (24.2) [6] | 6.9 (47.2) [6] | - | - | - | - |
| **Day 8^a^ (%)** | - | - | - | 0.2 (27.4) [6] | 0.2 (49.0) [6] | 0.2 (18.2) [6] |
| **Day 15^a^ (%)** | - | - | 0.3 (26.4) [6] | 0.4 (12.1) [5] | 0.3 (45.5) [6] | 0.3 (12.4) [6] |
| **Day 29^a^ (%)** | 0.7 (33.0) [3] | 0.5 (50.1) [6] | - | - | - | - |

^a^As percentage of serum concentration

Data are presented as geometric mean (CV %) [n]

**CSF**, cerebrospinal fluid; **CV**, coefficient of variation; **IV**, intravenous; **n**, number of subjects with valid observations

**Supplementary Table 8.** Summary of the serum pharmacokinetic parameters for MEDI1341 following single IV Doses of MEDI1341 in healthy participants

|  | **70 mg (N=6)** | **210 mg (N=6)** | **400 mg (N=6)** | **1200 mg (N=6)** | **2400 mg (N=6)** | **4500 mg (N=6)** |
| --- | --- | --- | --- | --- | --- | --- |
| AUC_0-t_ | 57.0 (16.9) [6] | 214 (25.0) [6] | 395 (32.1) [6] | 1080 (19.5) [6] | 2510 (10.1) [6] | 7460 (15.3) [6] |
| AUC_0-28d_ (day*µg/mL) | 52.3 (19.0) [6] | 184 (21.5) [6] | 317 (41.1) [6] | 875 (26.6) [6] | 2150 (8.2) [6] | 5800 (14.9) [6] |
| AUC_0-∞_ (day*µg/mL) | 64.1 (14.6) [6] | 222 (24.2) [6] | 407 (32.2) [6] | 1110 (19.8) [6] | 2590 (11.3) [6] | 7630 (15.3) [6] |
| C_max_ (µg/mL) | 11.5 (30.4) [6] | 43.1 (19.3) [6] | 46.3 (122.1) [6] | 149 (96.1) [6] | 462 (9.2) [6] | 1130 (13.4) [6] |
| t_max_^a^ (h) | 0.983  (0.9–9.0) [6] | 0.975  (0.9–9.0) [6] | 17.0  (0.9–67.3) [6] | 9.06  (1.0–72.9) [6] | 0.983  (1.0–9.0) [6] | 5.01  (1.0–9.0) [6] |
| t_last_^a^ (day) | 41.9  (27.7–56.0) [6] | 56.0  (55.8–91.1) [6] | 90.8  (55.8–91.1) [6] | 90.9  (55.7–91.2) [6] | 90.7  (88.8–93.1) [6] | 90.3  (88.7–91.0) [6] |
| t_1/2λz_ (day) | 17.4 (27.1) [6] | 16.6 (16.9) [6] | 17.3 (13.2) [6] | 17.3 (49.1) [6] | 24.3 (20.8) [6] | 18.9 (13.2) [6] |
| MRT (day) | 15.0 (33.6) [6] | 14.4 (18.9) [6] | 17.6 (33.0) [6] | 17.1 (40.0) [6] | 16.4 (17.9) [6] | 18.8 (12.0) [6] |
| CL (L/day) | 1.1 (14.6) [6] | 0.9 (24.2) [6] | 1.0 (32.2) [6] | 1.1 (19.8) [6] | 0.9 (11.3) [6] | 0.6 (15.3) [6] |
| V_ss_ (L) | 16.3 (40.4) [6] | 13.6 (19.3) [6] | 17.3 (63.1) [6] | 18.5 (46.8) [6] | 15.2 (8.8) [6] | 11.1 (17.9) [6] |
| V_z_ (L) | 27.4 (33.9) [6] | 22.6 (19.6) [6] | 24.5 (39.9) [6] | 27.0 (42.6) [6] | 32.6 (19.7) [6] | 16.0 (19.9) [6] |

Data are presented as geometric mean (CV%) [number of participants with valid observations]

^a^Median (min-max) [number of participants with valid observations]

**AUC_0-t_,** area under the concentration-time curve from time 0 to the time of the last quantifiable concentration; **AUC_0-28d_**, area under the concentration-time curve from time zero to 28 days postdose; **AUC_0-∞_**_,_ area under the concentration time curve from time zero to infinity; **CL**, total serum clearance; **C_max_**, maximum observed concentration; **CV**, coefficient of variation; **IV**, intravenous; **MRT**, mean residence time; **t_1/2λz_**, terminal elimination half-life; **t_last_**, time of last quantifiable concentration; **t_max_**, time to maximum observed concentration; **V_ss_**, volume of distribution at steady-state; **V_z_**, volume of distribution during the terminal elimination phase.

**Supplementary Table 9.** Statistical assessment of dose proportionality of the serum pharmacokinetic parameters of MEDI1341 following the first (Day 1) and third (Day 57) dose of MEDI1341 in participants with Parkinson’s disease (ANOVA model)

|  |  |  |  |  | **Pairwise p-value** | |
| --- | --- | --- | --- | --- | --- | --- |
| **Timepoint** | **Parameter** | **Dose** | **n** | **GLSM** | **1200 mg**  **(n=9)** | **2000 mg**  **(n=9)** |
| **Day 1** | **DAUC_0–τ_ (µg*day/mL/mg)** | **1200 mg** | 9 | 0.986 | - | 0.3271 |
|  |  | **2000 mg** | 8 | 1.08 | 0.3271 | - |
|  | **DC_max_ (µg/mL/mg)** | **1200 mg** | 9 | 0.211 | - | 0.8401 |
|  |  | **2000 mg** | 8 | 0.215 | 0.8401 | - |
| **Day 57** | **DAUC_0 –τ_ (µg*day/mL/mg)** | **1200 mg** | 8 | 1.57 | - | 0.6317 |
|  |  | **2000 mg** | 8 | 1.50 | 0.6317 | - |
|  | **DC_max_ (µg/mL/mg)** | **1200 mg** | 8 | 0.214 | - | 0.4889 |
|  |  | **2000 mg** | 8 | 0.233 | 0.4889 | - |

The estimates of GLSMs were obtained by taking the exponential of the corresponding estimates of LSMs on the natural log (ln) scale

**AUC_0–τ_**, area under the concentration-time curve from time zero to the end of the dosing interval; **C_max_**, maximum observed concentration; **DAUC_0–τ_**, AUC_0–τ_ normalized by dose administered; **DC_max_**, C_max_ normalized by dose administered; **GLSM**, geometric least squares mean; **IV**, intravenous; **LSM**, least squares mean; **n**, number of subjects with valid observations

**Supplementary Table 10.** CSF MEDI1341 concentrations and CSF/serum concentration ratios following multiple IV doses of MEDI1341

| **Timepoint** | **MEDI1341 1200 mg (N=7)** | **MEDI1341 2000 mg**  **(N=7)** |
| --- | --- | --- |
| **Predose (ng/mL)** | - | - |
| **Day 61 (ng/mL)** | 250 (22.1) [7] | 329 (33.3) [6] |
| **Day 85 (ng/mL)** | 122 (23.3) [7] | 181 (42.8) [7] |
| **Day 61^a^ (%)** | 0.2 (33.6) [7] | 0.2 (73.6) [6] |
| **Day 85^a^ (%)** | 0.5 (41.6) [7] | 0.6 (99.8) [7] |

^a^As percentage of serum concentration

Data are presented as geometric mean (CV%) [n]

**CSF**, cerebrospinal fluid; **CV**, coefficient of variation; **IV**, intravenous; **n**, number of subjects with valid observations

**Supplementary Table 11.** Summary of the serum pharmacokinetic parameters for MEDI1341 following the first intravenous dose of MEDI1341 in participants with Parkinson’s disease

| **Parameter** | **1200 mg (N=9)** | **2000 mg (N=9)** |
| --- | --- | --- |
| **AUC_0-τ_ (µg*day/mL)** | 1180 (15.3) [9] | 2160 (22.5) [8] |
| **AUC_0-t_ (µg*day/mL)** | 1190 (14.2) [9] | 2210 (21.4) [9] |
| **C_max_ (µg/mL)** | 253 (15.4) [9] | 429 (24.3) [8] |
| **t_max_ (h)** | 1.08 (0.97–25.4) [9] | 1.05 (0.93–50.4) [9] |
| **t_last_ (day)** | 28.0 (25.0–33.0) [9] | 28.0 (20.1–33.0) [9] |

Data are presented as geometric mean (CV%)
**AUC_0-t_**, area under the concentration-time curve from time 0 to the time of the last quantifiable concentration (t_last_); **AUC_0-τ_**, area under the concentration-time curve from time zero to the end of the dosing interval; **C_max_**, maximum observed concentration; **CV**, coefficient of variation (%); **n**, number of subjects with valid observations; **t_last_**, time of last quantifiable concentration; **t_max_**, time to maximum observed concentration

**Supplementary Table 12.** Summary of the serum pharmacokinetic parameters for MEDI1341 following the third intravenous dose of MEDI1341 in participants with Parkinson’s disease

| **Parameter** | **1200 mg (N=9)** | **2000 mg (N=9)** |
| --- | --- | --- |
| **AUC_0-τ_ (µg*day/mL)** | 1890 (19.1) [8] | 2990 (22.0) [8] |
| **AUC_0-t_ (µg*day/mL)** | 2480 (21.2) [8] | 3800 (25.0) [8] |
| **C_max_ (µg/mL)** | 257 (18.7) [8] | 465 (29.4) [8] |
| **t_max_ (h)** | 1.1 (1.0–25.1) [8] | 1.0 (0.9–9.1) [8] |
| **t_last_ (day)** | 90.5 (84.1–97.0) [8] | 91.1 (85.1–98.1) [8] |
| **t_1/2λz_ (day)** | 18.2 (29.6) [7] | 19.5 (10.4) [7] |
| **CL (L/day)** | 0.636 (19.1) [8] | 0.669 (22.0) [8] |
| **V_z_ (L)** | 16.4 (38.3) [7] | 19.2 (21.2) [7] |
| **R_o_** | 1.61 (19.6) [8] | 1.35 (14.5) [7] |

Data are presented as geometric mean (CV%) [number of participants with valid observations]

^a^Median (min-max) [number of participants with valid observations]

**AUC_0-τ_ ,** area under the concentration-time curve from time 0 to the end of the dosing interval; **AUC_0-t_**_,_ area under the concentration-time curve from time zero to the time of the last quantifiable concentration; **CL**, total serum clearance; **C_max_**, maximum observed concentration; **CV**, coefficient of variation; **R_o_,** accumulation ratio = AUC_0-τ_ (last infusion)**/**AUC_0-τ_ (first infusion); ; **t_1/2λz_**, terminal elimination half-life; **t_last_**, time of last quantifiable concentration; **t_max_**, time to maximum observed concentration; **V_z_**, volume of distribution during the terminal elimination phase

**Supplementary Table 13.** Percentage change from baseline in CSF free α-synuclein concentration following single MEDI1341 IV dose in healthy participants

| **Treatment** | **Day 8** | | **Day 15** | | **Day 29** | |
| --- | --- | --- | --- | --- | --- | --- |
|  | **Mean (SD)** | **95% CI** | **Mean (SD)** | **95% CI** | **Mean (SD)** | **95% CI** |
| Placebo | –0.2 (20.2) | –25.3, 24.8 | –3.8 (15.4) | –18.1, 10.5 | –5.3 (9.0) | –16.5, 5.9 |
| MEDI1341 70 mg | – | – | – | - | –3.9 (6.7) | –11.0, 3.1 |
| MEDI1341 210 mg | – | – | – | - | –2.8 (9.7) | –13.1, 7.4 |
| MEDI1341 400 mg | – | – | –23.7 (5.7) | –29.7, 17.6* | – | – |
| MEDI1341 1200 mg | –42.9 (12.3) | –55.8, –30.0* | –25.3 (20.4) | –50.6, –0.0* | –25.5 | – |
| MEDI1341 2400 mg | –45.0 (8.8) | –54.2, –35.7* | –28.5 (8.8) | –37.6, -19.3* | – | – |
| MEDI1341 4500 mg | –47.6 (27.2) | –76.2, –19.0* | –53.7 (9.2) | -63.3, -44.1* | – | – |
|  | **Median** | **Range** | **Median** | **Range** | **Median** | **Range** |
| Placebo | –11.3 | –16.0, 29.3 | –4.9 | –23.1, 24.8 | –3.4 | –18.3, 4.7 |
| MEDI1341 70 mg | – | – | – | – | –5.3 | –9.9, 7.2 |
| MEDI1341 210 mg | – | – | – | – | –2.8 | –17.0, 8.1 |
| MEDI1341 400 mg | – | – | –21.4 | –34.7, – 19.4 | – | – |
| MEDI1341 1200 mg | –42.7 | –59.9, –27.2 | –31.1 | –51.3, 1.5 | – | – |
| MEDI1341 2400 mg | –46.3 | –55.5, –30.0 | –30.2 | –38.3, –17.9 | – | – |
| MEDI1341 4500 mg | –58.3 | –62.3, 7.8 | –53.6 | –64.5, –43.3 | – | – |

**CSF**, cerebrospinal fluid; **IV**, intravenous; **SD**, standard deviation

*Pairwise comparison with placebo using Welch’s t-test p <0.01.

**Supplementary Table 14. Percentage c**hange from baseline in CSF free α-synuclein concentration following multiple MEDI1341 IV doses in participants with Parkinson’s disease

|  | **Day 61**  **(Day 4 after 3^rd^ infusion)** | | **Day 85**  **(Day 28 after 3^rd^ infusion)** | |
| --- | --- | --- | --- | --- |
|  | **Mean (SD)** | **95% CI** | **Mean (SD)** | **95% CI** |
| Placebo | 11.3 (52.2) | –37.0, 59.6 | 0.5 (33.8) | -30.8, 31.7 |
| MEDI1341 1200mg | –44.9 (32.2) | –74.7, -15.1 | –46.2 (20.1) | -64.8, -27.6 |
| MEDI1341 2000 mg | –64.4 (26.1) | –91.8,-37.0 | –59.3 (14.6) | -72.8, -45.8 |
|  | **Median** | **Range** | **Median** | **Range** |
| Placebo | –7.1 | –27.4, –127.0 | 12.6 | –36.5, 35.8 |
| MEDI1341 1200mg | –55.5 | –77.3, –7.4 | –51.5 | –66.9, –17.3 |
| MEDI1341 2000 mg | –75.2 | –81.9, –13.0 | –59.0 | –80.3, –37.6 |

**CI**, confidence interval; **CSF**, cerebrospinal fluid; **IV**, intravenous; **SD**, standard deviation

**Supplementary Table 15**. Summary of Montreal Cognitive Assessment (Total Score) in participants with Parkinson’s disease

|  | **Placebo**  **(N=7)** | **1200 mg**  **(N=9)** | **2000 mg**  **(N=9)** | **Overall**  **(N=25)** |
| --- | --- | --- | --- | --- |
| **Day -1** | 27.7 (1.5) [7] | 27.2 (2.1) [9] | 27.8 (1.0) [9] | 27.6 (1.5) [25] |
| **Day 85** | 26.9 (2.5) [7] | 28.1 (3.0) [8] | 28.2 (1.5) [9] | 27.8 (2.4) [24] |
| **Day 148** | 27.3 (1.8) [7] | 27.0 (3.4) [8] | 27.1 (2.3) [9] | 27.1 (2.5) [24] |

Data are presented as mean (SD) [n]

**n**, number of subjects assessed at timepoint; **SD**, standard deviation

**Supplementary Table 16**. Summary of Movement Disorder Society – Unified Parkinson’s Disease Rating Scale (total score) in participants with Parkinson’s disease

|  | **Placebo**  **(N=7)** | **1200 mg**  **(N=9)** | **2000 mg**  **(N=9)** |
| --- | --- | --- | --- |
| **Part I (Non-Motor Aspects of Experiences of Daily Living)** | | | |
| Screening | 4.1 (3.1) [7] | 7.7 (3.8) [9] | 6.3 (5.9) [9] |
| Day 85 | 3.3 (3.0) [7] | 7.4 (4.3) [8] | 6.6 (4.5) [9] |
| Day 148 | 5.0 (3.5) [7] | 7.1 (4.7) [8] | 7.0 (4.7) [9] |
| **Part II (Motor Aspects of Experiences of Daily Living)** | | | |
| Screening | 6.4 (4.3) [7] | 11.2 (6.2) [9] | 6.2 (5.5) [9] |
| Day 85 | 6.3 (4.0) [7] | 9.3 (5.7) [8] | 7.4 (6.4) [9] |
| Day 148 | 8.0 (6.1) [7] | 9.1 (5.5) [8] | 8.3 (6.6) [9] |
| **Part III (Motor Examination)** | | | |
| Screening | 28.3 (15.2) [7] | 27.7 (12.7) [9] | 20.7 (7.0) [9] |
| Day 85 | 31.4 (13.9) [7] | 27.5 (16.4) [8] | 22.6 (12.5) [9] |
| Day 148 | 26.3 (15.8) [7] | 24.4 (14.9) [8] | 23.6 (10.6) [9] |
| **Part IV (Motor Complications)** | | | |
| Screening | 3.6 (2.5) [7] | 3.1 (2.4) [9] | 3.2 (5.2) [9] |
| Day 85 | 4.7 (2.5) [7] | 3.8 (2.8) [8] | 2.3 (4.3) [9] |
| Day 148 | 2.7 (2.1) [7] | 2.5 (2.4) [8] | 3.1 (3.7) [9] |
| **Total modified** |  |  |  |
| Screening | 42.4 (18.0) [7] | 49.7 (14.3) [9] | 36.4 (16.9) [9] |
| Day 85 | 45.7 (13.9) [7] | 47.9 (23.0) [8] | 38.9 (21.0) [9] |
| Day 148 | 42.0 (21.4) [7] | 43.1 (20.3) [8] | 42.0 (18.7) [9] |

Data are presented as mean (SD) [n]

**n**, number of participants assessed at timepoint; **SD**, standard deviation
